# Low production of 12α-hydroxylated bile acids prevents hepatic steatosis in *Cyp2c70*^−/−^ mice by reducing fat absorption

**DOI:** 10.1016/j.jlr.2021.100134

**Published:** 2021-10-07

**Authors:** Rumei Li, Anna Palmiotti, Hilde D. de Vries, Milaine V. Hovingh, Martijn Koehorst, Niels L. Mulder, Yue Zhang, Kim Kats, Vincent W. Bloks, Jingyuan Fu, Henkjan J. Verkade, Jan Freark de Boer, Folkert Kuipers

**Affiliations:** 1 Department of Pediatrics, University of Groningen, University Medical Center Groningen, Groningen, The Netherlands; 2 Department of Laboratory Medicine, University of Groningen, University Medical Center Groningen, Groningen, The Netherlands; 3 Department of Genetics, University of Groningen, University Medical Center Groningen, Groningen, The Netherlands; 4 Department of Biomedical Science of Cells and Systems, University of Groningen, University Medical Center Groningen, Groningen, The Netherlands

**Keywords:** bile acids, *Cyp2c70*, *Cyp8b1*, fat absorption, obesity, fatty liver disease, humanized mouse model, BA, bile acid, BAT, brown adipose tissue, BSTFA, N,O-bis(trimethylsilyl) trifluoroacetamide, BW, body weight, CA, cholic acid, CDCA, chenodeoxycholic acid, CK19, cytokeratin 19, *Cyp2c70*, cytochrome P450, family 2, subfamily C, polypeptide 70, DCA, deoxycholic acid, MCA, muricholic acid, NAFLD, nonalcoholic fatty liver disease, NASH, nonalcoholic steatohepatitis, OTU, operational taxonomic unit, PL, phospholipid, scWAT, subcutaneous white adipose tissue, TEM, transmission electron microscopy, TGR5, Takeda G protein-coupled receptor 5, WTD, Western-type high-fat diet

## Abstract

Bile acids (BAs) play important roles in lipid homeostasis, and BA signaling pathways serve as therapeutic targets for nonalcoholic fatty liver disease (NAFLD). Recently, we generated cytochrome P450, family 2, subfamily C, polypeptide 70 (*Cyp2c70*^−/−^) mice with a human-like BA composition lacking mouse-/rat-specific muricholic acids to accelerate translation from mice to humans. We employed this model to assess the consequences of a human-like BA pool on diet-induced obesity and NAFLD development. Male and female *Cyp2c70*^−/−^ mice and WT littermates were challenged with a 12-week high-fat Western-type diet (WTD) supplemented with 0.25% cholesterol. *Cyp2c70* deficiency induced a hydrophobic BA pool with high abundances of chenodeoxycholic acid, particularly in females, because of sex-dependent suppression of sterol 12α-hydroxylase (*Cyp8b1*). Plasma transaminases were elevated, and hepatic fibrosis was present in *Cyp2c70*^−/−^ mice, especially in females. Surprisingly, female *Cyp2c70*^−/−^ mice were resistant to WTD-induced obesity and hepatic steatosis, whereas male *Cyp2c70*^−/−^ mice showed similar adiposity and moderately reduced steatosis compared with WT controls. Both intestinal cholesterol and FA absorption were reduced in *Cyp2c70*^−/−^ mice, the latter more strongly in females, despite unaffected biliary BA secretion rates. Intriguingly, the biliary ratio 12α-/non-12α-hydroxylated BAs significantly correlated with FA absorption and hepatic triglyceride content as well as with specific changes in gut microbiome composition. The hydrophobic human-like BA pool in *Cyp2c70*^−/−^ mice prevents WTD-induced obesity in female mice and NAFLD development in both genders, primarily because of impaired intestinal fat absorption. Our data point to a key role for 12α-hydroxylated BAs in control of intestinal fat absorption and modulation of gut microbiome composition.

Nonalcoholic fatty liver disease (NAFLD), also referred to as metabolic-associated fatty liver disease (1), is an increasingly common cause of chronic liver disease and an important component of a cluster of cardiometabolic diseases that includes obesity, hyperlipidemia, and insulin resistance. NAFLD, primarily characterized by hepatic steatosis, may further develop into nonalcoholic steatohepatitis (NASH) and progress to cirrhosis and hepatocellular carcinoma (2). The pathogenesis of NAFLD and the determinants of its progression to NASH remain poorly understood, and effective treatment strategies are urgently needed. Over the past years, relationships between bile acid (BA) metabolism and various aspects of NAFLD have been identified. Patients with obesity and NAFLD/NASH exhibit higher fasting and postprandial plasma BA concentrations, which in case of NASH conceivably relates to coexistent insulin resistance (3). Moreover, BAs and BA signaling pathways have been reported to regulate lipid, glucose, and energy homeostasis as well as inflammatory responses (4) and thereby impact disease development. Based on this knowledge, BA signaling pathways acting through the FXR/NR1H4 or Takeda G protein-coupled receptor 5 (TGR5/G protein-coupled BA receptor 1) have been identified as potential targets for NAFLD/NASH treatment (5). In addition, “classical functions” of BAs in absorption of dietary fats and fat-soluble vitamins, turnover of hepatic cholesterol, regulation of biliary lipid secretion (6), and control of microbiome composition (7) may also impact hepatic lipid homeostasis.

Differently structured BAs that comprise the human BA pool display a wide variation in lipid-solubilizing capacity as well as in FXR/TGR5-activating potency (6), and size and composition of this pool show large interindividual variations, even in healthy subjects (8, 9). It is important to realize that mechanistic preclinical studies addressing the roles of BAs in the etiology of obesity and NAFLD/NASH have been performed in mice and rats in which BA composition fundamentally differs from that in humans. This evidently complicates extrapolation of the outcomes of animal experiments to human situations. While cholic acid (CA; 3α,7α,12α-trihydroxy-5β-cholan-24-oic acid), chenodeoxycholic acid (CDCA; 3α,7α-dihydroxy-5β-cholan-24-oic acid), and deoxycholic acid (DCA; 3α,12α-dihydroxy-5β-cholan-24-oic acid) are predominant BA species in humans, CA and muricholic acids (MCAs; αMCA, βMCA, and ωMCA, i.e., 3α, 6β, 7α-, 3α, 6β, 7β-, and 3α, 6α, 7β-trihydroxy-5β-cholan-24-oic acid, respectively) are predominant BAs in mice and rats. The very hydrophilic MCAs are synthesized from hydrophobic CDCA and have poor lipid-solubilizing capacities and act as FXR antagonists rather than agonists (10). Generally, more hydrophobic BAs are superior in micelle formation as well as in activation of the various BA receptors (6). Since cytochrome P450, family 2, subfamily C, polypeptide 70 (CYP2C70) was reported to mediate MCA formation (11), *Cyp2c70*^−/−^ mice have been generated by us (12) and by others (13, 14), showing a human-like BA composition with high abundances of CDCA and absence of MCAs.

We have recently reported that hepatic BA synthesis and pool size are reduced in *Cyp2c70*^−/−^ mice (12), particularly in females (12), indicating that the presence of MCAs might explain the relatively high BA synthesis rate in mice compared with humans (15). It is currently not known how introduction of a human-like BA pool modulates diet-induced NALFD development in mice. Therefore, we fed *Cyp2c70*^−/−^ mice with a Western-type diet (WTD) for 12 weeks and observed that the absence of MCAs was associated with low levels of 12α-hydroxylated BAs (CA and DCA), particularly in female mice. Surprisingly, female *Cyp2c70*^−/−^ mice were protected from diet-induced obesity and hepatic steatosis. Our data show that the metabolic resistance to WTD in *Cyp2c70*^−/−^ mice is mainly driven by a modest reduction of dietary fat absorption, likely attributable to the strongly reduced contribution of 12α-hydroxylated BAs to the circulating BA pool.

## Materials and methods

### Animals

The C57BL/6J-Cyp2c70^em3Umcg^ (*Cyp2c70*^−/−^) mice (12) and their WT littermates were bred in the animal facility of the University Medical Center Groningen and housed in a temperature-controlled room (21°C) with a 12-h light/12-h dark cycle and ad libitum access to food and water. All animal experiments were approved by the Dutch Central Committee for Animal Experiments and the Animal Welfare Body of the University of Groningen and received humane care according to the criteria outlined in the Guide for the Care and Use of Laboratory Animals (16). Individually housed male and female *Cyp2c70*^−/−^ mice (male = 8 and female = 8) and WT littermates (male = 7 and female = 10) received a WTD with 60% (energy) fat and 0.25% cholesterol (D14010701Bi; Research Diets, Inc, New Brunswick, NJ) for 12 weeks, starting at the age of 12–14 weeks. Body weights (BWs) and food intake were monitored weekly. Immediately before start of the WTD and after 11 weeks of WTD feeding, noninvasive measurements of body composition (fat mass, lean mass, and fluid mass) were made in conscious mice, using a Minispec Body Composition Analyzer (LF90; Bruker BioSpin GmbH, Rheinstetten, Germany), and oral glucose tolerance tests were performed. Blood and feces samples were collected after the diet intervention. After 12 weeks of WTD feeding, mice were fasted for 4 h (08:00–12:00 h) and euthanized through cardiac puncture under isoflurane anesthesia. Plasma and organs were collected and stored at −80°C until analysis.

### Assessment of glucose tolerance

Glucose tolerance tests were performed immediately before start of WTD feeding and at 11 weeks of WTD feeding. After 4 h of fasting (08:00–12:00 h), mice received a glucose bolus (2 g/kg BW) by oral gavage. Blood glucose levels were measured before and at 5, 15, 30, 45, 60, 90, and 120 min after gavage using a handheld glucose meter (Accu-Chek Performa; Roche Diabetes Care, Almere, the Netherlands). Before and at 5, 15, 60, and 120 min after injection, small blood spots (10 μl) were taken by tail tip bleeding on filter paper (Satorius Stedim TFN 180 g/m^2^; Nieuwegein, the Netherlands) for insulin measurement (17). Blood spots were briefly air dried and stored at −20°C until extraction and measurement of insulin (Crystal Chemrat insulin ELISA [catalog no. 90010] kit with mouse insulin standard [catalog no. 90020], Zaandam, the Netherlands).

### Intestinal lipid uptake

To visualize intestinal FA uptake, separate cohorts of 8-week WTD-fed mice were fasted overnight to remove existing intestinal lipid stores and then received an oral gavage with olive oil (200 μl/25 g BW) containing 0.2 μg/μl BODIPY-labeled palmitic acid (BODIPY™ FL C16, D3821; Thermo Fisher Scientific, Inc, MA) (18). Two hours after gavage, mice were sacrificed, and intestines were immediately excised and fixed in OCT compound. Sections (4 μm) were cut and mounted in ProLong® Diamond Antifade Mountant mounting medium containing 4,6-diamidino-2-phenylindole and were analyzed by fluorescence microscopy. Images were obtained using a Leica DMI6000B fluorescence microscope (Leica Microsystems, Wetzlar, Germany) equipped with a Leica DFC365 FX camera and LAS AF software.

### Electron microscopy

Large-scale electron microscopy (“nanotomy”) was performed using standard protocols as previously described (19). Briefly, primary fixation was performed in 2% glutaraldehyde and 2% paraformaldehyde in 0.1 M cacodylate buffer (pH 7.4). Cells were osmicated prior to embedding with 1.5% osmium tetroxide/potassium ferrocyanide (20). Ultrathin (80 nm) sections were placed on single slot (2 × 1 mm) copper grids and contrasted with neodymium (21). Acquisition was on a Zeiss Supra55 ATLAS following procedures described (19).

### Gallbladder cannulations

In different groups of WTD-fed mice, gallbladder cannulations were performed to evaluate bile formation (12). Mice were anesthetized with Hypnorm (fentanyl/fluanisone; 1 ml/kg) and diazepam (10 mg/kg) after which the bile duct was ligated and the gallbladder cannulated. Mice were then placed into a humidified incubator to maintain body temperature, and bile was collected continuously for 30 min. Bile production was determined gravimetrically.

### Biliary cholesterol and biliary phospholipid analyses

Biliary cholesterol (12) and phospholipid (PL) (22) concentrations were measured as described following lipid extraction according to Bligh and Dyer (23).

### BA analyses

LC-tandem mass spectrometry was applied for quantification of individual BA species in plasma and bile using a Nexera X2 Ultra High Performance Liquid Chromatography system (SHIMADZU, Kyoto, Japan), coupled to a SCIEX QTRAP 4500 MD triple quadrupole mass spectrometer (SCIEX, Framingham, MA) (ultrahigh performance liquid chromatography-MS/MS), as previously described (24).

For quantification of fecal BAs, feces were collected from individual mice for 72 h, dried, and thoroughly ground. About 50 mg of dried feces was incubated in alkaline methanol at 80°C for 2 h. BAs were purified using C18 Sep-Pak columns (Waters, Milford, MA), methylated with methanol/acetyl chloride in a ratio 20:1, and derivatized with pyridine/N,O-bis(trimethylsilyl) trifluoroacetamide (BSTFA)/trimethylchlorosilane (50:50:1) for 1 h. Samples were then dried at room temperature under a stream of nitrogen and dissolved in heptane containing 1% BSTFA for quantification by GC using 5β-cholanic acid-7α,12α-diol as internal standard (25).

### Hepatic lipid analyses

Hepatic triglycerides and total cholesterol were measured using commercially available reagents (Roche Diagnostics [Rotkreuz, Switzerland] and DiaSys Diagnostic Systems [Holzheim, Germany], respectively) following lipid extraction from liver homogenates as described by Bligh and Dyer (23).

### Fecal neutral sterol analyses

For quantification of fecal neutral sterol excretion, feces were collected from individual mice for 72 h, dried, and thoroughly ground. About 50 mg of dried feces was incubated in alkaline methanol at 80°C for 2 h. Neutral sterols were then extracted three times with petroleum ether (boiling range 60–80°C) and derivatized with pyridine/BSTFA/trimethylchlorosilane (50:50:1) for 1 h. Samples were then dried at room temperature under a stream of nitrogen and dissolved in heptane containing 1% BSTFA for quantification by GC using 5a-cholestane standard (25).

### Determination of fecal energy content

Samples of ∼300 mg powdered dry feces were combusted in a Parr 6100 compensated calorimeter (Parr Instrument Company, Moline, IL) using a 1108 Oxygen Bomb placed in 2,000 g of demineralized water. The caloric content of the feces was derived from the temperature increase of the water. The intra-assay variability was ∼0.3%.

### Plasma biochemistry

Plasma triglycerides (Roche Diagnostics), total cholesterol, free cholesterol, nonesterified FAs (all DiaSys Diagnostic Systems) were quantified using commercially available reagents.

Plasma lipoproteins were separated by fast protein liquid chromatography using a system containing a PU-4180 pump with a linear degasser and UV-4075 UV/VIS detectors (Jasco, Tokyo, Japan), as described (12). Plasma plant sterols were determined by GC as described (26). Circulating levels of aspartate aminotransferase, alanine aminotransferase, and albumin were determined using a routine clinical chemistry analyzer (Cobas 6000; Roche Diagnostics) with standard reagents (Roche Diagnostics).

### Gene expression analysis

Total RNA was isolated from liver and small intestine using TRI reagent (Sigma, St. Louis, MO), quantified by NanoDrop (NanoDrop Technologies, Wilmington, DE) and reverse transcribed using Moloney-Murine Leukemia Virus reverse transcriptase (Life Technologies, Bleiswijk, the Netherlands). RNA from brown adipose tissue (BAT) and subcutaneous white adipose tissue (scWAT) was isolated using an RNeasy Lipid Tissue Mini Kit (Qiagen, Venlo, the Netherlands) quantified by NanoDrop and reverse transcribed as detailed apreviously. Real-time quantitative PCR analyses were performed on a Step One Plus™ Real-Time PCR system (Applied Biosystems, Foster City, CA). Gene expression levels were normalized to *cyclophilin* as a housekeeping gene for liver and intestine and *36B4* (*Rplp0*) as a housekeeping gene for BAT and scWAT. Data were then further normalized to the mean of the respective control group.

### Histology

After termination, tissues were rapidly excised and fixed in 4% formalin for 24 h prior to embedding in paraffin. Sections (4 μm) were cut and stained with hematoxylin and eosin or Sirius Red/Fast Green dye combination. Immunohistochemical staining for cholangiocytes was performed using an anti-cytokeratin 19 (CK19) antibody (ab52625; Abcam, Cambridge, United Kingdom) according to the manufacturer's instructions. Images were acquired using a Hamamatsu NanoZoomer (Hamamatsu Photonics, Almere, the Netherlands).

### Microbiome analysis

DNA was extracted from cecal contents of both male and female *Cyp2c70*^−/−^ mice and WT controls after 12 weeks of WTD feeding. 16S sequencing of V4 region was performed on an Illumina HiSeq platform (Novogene, Hong Kong, China) as previously described (1). Briefly, an operational taxonomic unit (OTU) table with 1,502 OTUs was generated by screening OTUs in small subunit rRNA database (with 97% similarity) for species annotation at each taxonomic rank (threshold: 0.8–1.0) (13, 14). It was rarefied to the minimum read number of all samples (119,545 reads) for further analysis.

Alpha diversity of the microbial composition was assessed using Shannon index, whereas principal coordinate analysis was performed based on the Bray-Curtis distance of beta diversity. The differences of alpha and beta diversity between genotypes or sex were analyzed by the Mann-Whitney *U* test and the permutational multivariate ANOVA statistical test, respectively. All rarefied OTUs were classified into family level. The rare bacterial families, which were present in less than five mice, were removed, leaving 136 families in total. The Mann-Whitney *U* test was used to analyze differential relative abundance of families between *Cyp2c70*^−/−^ mice and WT controls with an adjusted *P* value for multiple comparisons using the Benjamini-Hochberg method. Differential abundances with an adjusted *P* < 0.1 were considered significant. Linear correlation analyses were performed between the relative abundance of *Erysipelotrichaceae* (%) and the biliary 12α/non-12α hydroxylated BA ratio, FA absorption efficiency, as well as hepatic total cholesterol levels. All statistical analyses for microbiome data were performed using R (version 4.0.5; R Foundation for Statistical Computing, Vienna, Austria).

### Statistical analysis

Data are presented as Tukey box-whisker plots or as line graphs with mean and SEM/SD as specified, which were generated using GraphPad Prism (version 8; Graph Pad Software, Inc, San Diego, CA). Significance of differences in multiple group comparisons were assessed using the Kruskal-Wallis H test, followed by Conover posthoc comparisons in BrightStat (BrightStat.com) (27), whereas the Mann-Whitney *U* test was used to compare *Cyp2c70*^−/−^ and WT mice within genders as specified. Differences with a *P* < 0.05 were considered statistically significant.

## Results

### Female but not male *Cyp2c70*^−/−^ mice are protected from WTD-induced obesity and insulin resistance

**Fig. 1:**
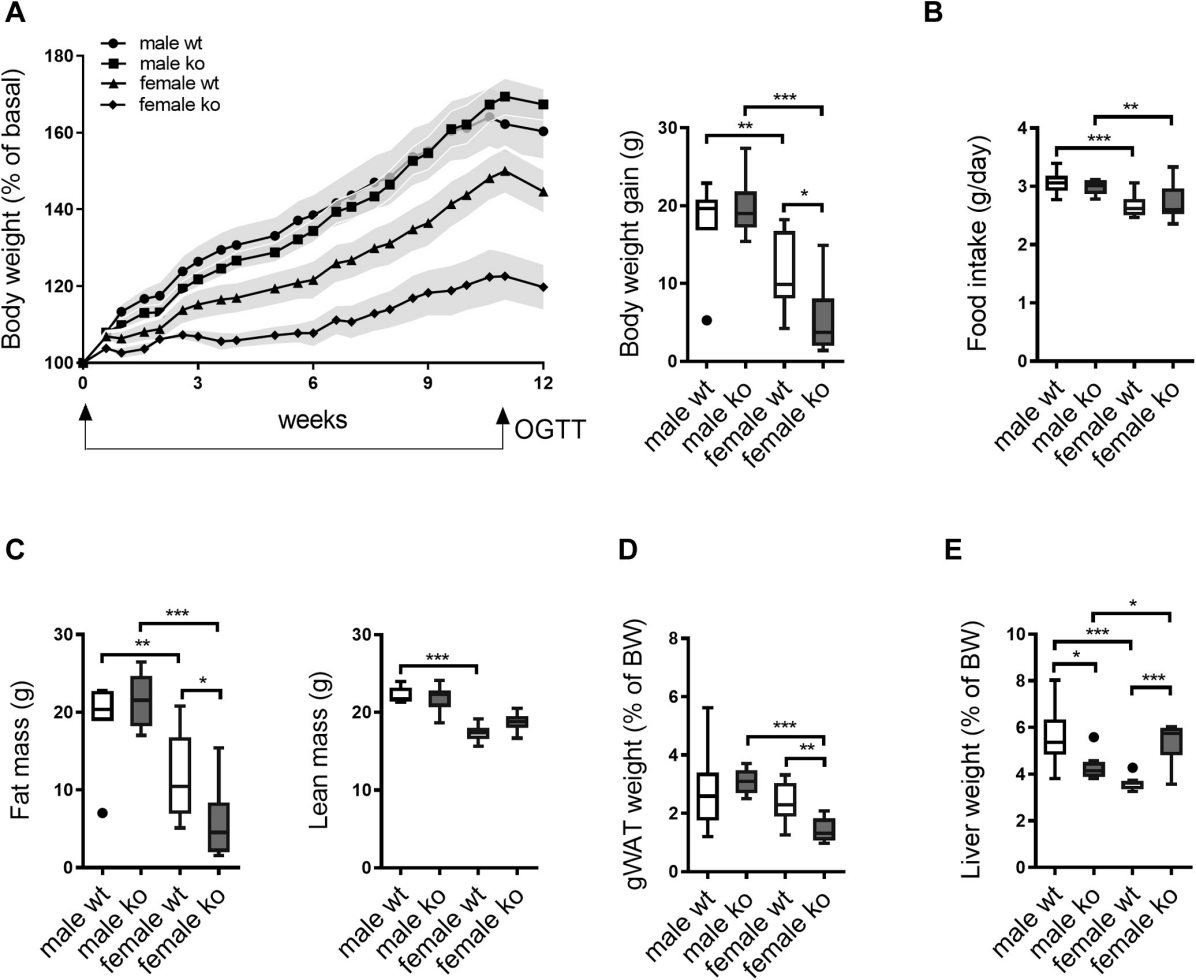
Female, but not male, *Cyp2c70*^−/−^ mice are protected from WTD-induced obesity despite unaffected food intake. A: BW development as percent of initial weight (data are presented as mean ± SEM) and as grams gained in male and female *Cyp2c70*^−/−^ mice and WT littermates during 12 weeks of WTD feeding. B: Average daily food intake in male and female *Cyp2c70*^−/−^ mice and WT littermates during 12 weeks of WTD feeding. C: Fat mass and lean mass in male and female *Cyp2c70*^−/−^ mice and WT littermates after 12 weeks of WTD feeding as determined by MiniSpec. D: gWAT weights as percent of BW in male and female *Cyp2c70*^−/−^ mice and WT littermates after 12 weeks of WTD feeding. E: Liver weights as percent of BW in male and female *Cyp2c70*^−/−^ mice and WT littermates after 12 weeks of WTD feeding. N = 7–10 mice/group. Data are presented as Tukey's box-and-whisker plots unless specified, and *P* values represent ∗*P* < 0.05, ∗∗*P* < 0.01, and ∗∗∗*P* < 0.001 by Kruskal-Wallis H testing followed by Conover posthoc comparisons. gWAT, gonadal white adipose tissue; OGTT, oral glucose tolerance test.

BW, fat mass, and lean mass of chow-fed 12-week-old *Cyp2c70*^+/+^ (WT) and *Cyp2c70*^−/−^ mice were similar in males and females (data not shown). Mice were then switched to the WTD to induce obesity and development of fatty liver. BW gain was reduced by 53% in female *Cyp2c70*^−/−^ mice compared with WT (Fig. 1A), despite identical food intake in the genotypes (Fig. 1B). Differences in BWs between the groups were entirely attributable to reduction of body fat (Fig. 1C, D). No differences in BW gain and adiposity were observed in male *Cyp2c70*^−/−^ mice (Fig. 1A–D), yet, liver weights were 49% higher in female *Cyp2c70*^−/−^ mice but 25% lower in male *Cyp2c70*^−/−^ mice than in WT controls (Fig. 1E).

No differences in fasting glucose levels were detected between *Cyp2c70*^−/−^ and WT mice before start of WTD in either gender (supplemental Table S1). Only female *Cyp2c70*^−/−^ mice did, however, display moderately improved glucose tolerance (supplemental Fig. S1A). Upon WTD intervention, only female *Cyp2c70*^−/−^ mice displayed lower fasting glucose and insulin levels and, hence, improved homeostatic model assessment-insulin resistance compared with controls (supplemental Fig. S1B, C and supplemental Table S1). Glucose tolerance tests performed after 11 weeks of WTD feeding revealed markedly better glucose handling in female but not in male *Cyp2c70*^−/−^ mice compared with WT (supplemental Fig. S1B, C). Collectively, these data indicate that insulin sensitivity is higher in WTD-fed female but not in male *Cyp2c70*^−/−^ mice. The reduced adiposity of female *Cyp2c70*^−/−^ mice conceivably contributed to the improved glucose handling.

### *Cyp2c70* deficiency impacts BA homeostasis most prominently in female mice

**Fig. 2:**
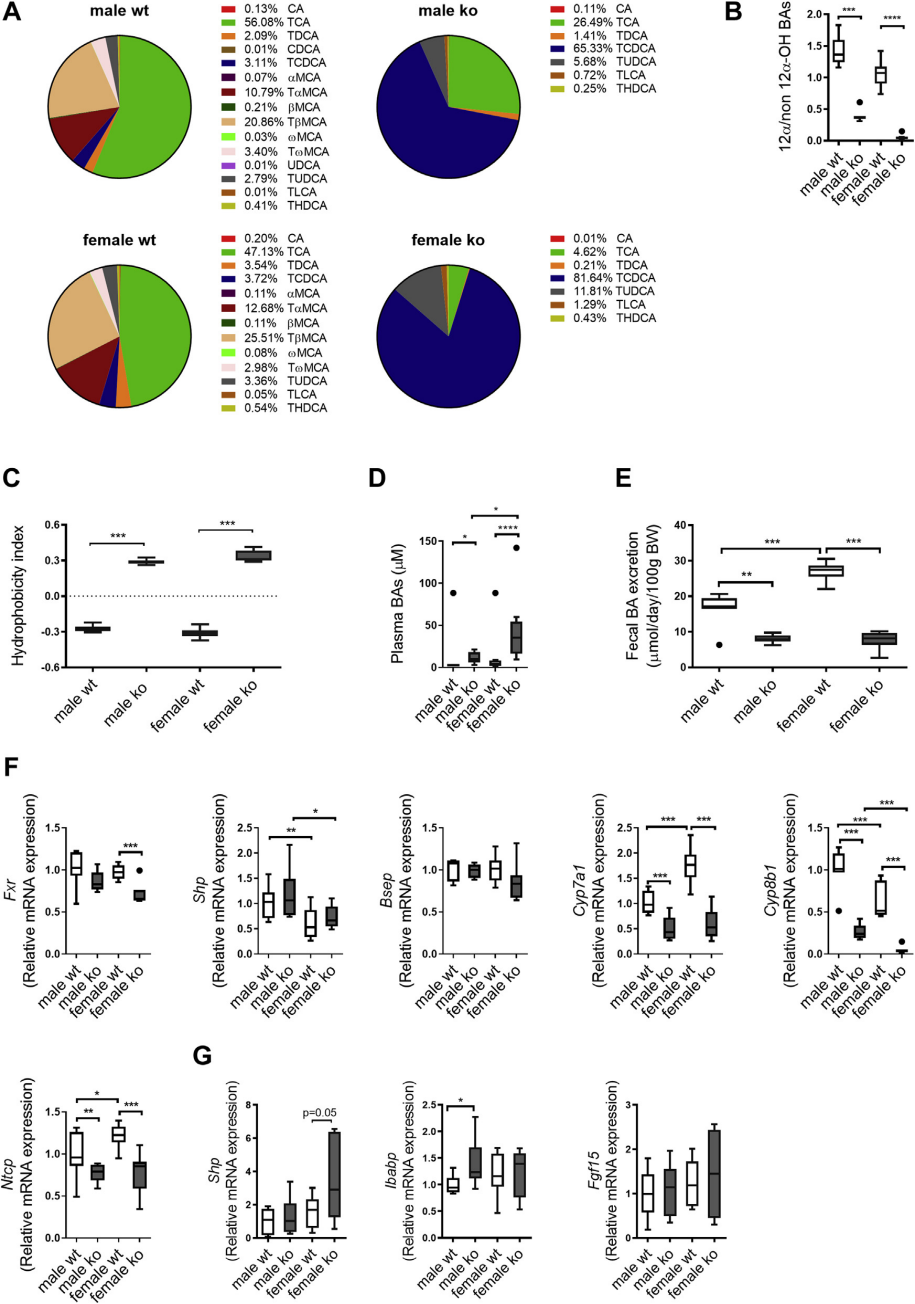
*Cyp2c70* deficiency induces a human-like hydrophobic bile acid pool in mice with a high abundance of chenodeoxycholic acid, particularly in females, and reduces hepatic bile acid synthesis. A: Bile acid composition and (B) ratio of 12α-/non-12α-hydroxylated BAs in gallbladder bile of male and female *Cyp2c70*^−/−^ mice and WT littermates after 12 weeks of WTD feeding. C: Hydrophobicity index of biliary bile acids. D: Bile acid concentrations in plasma of male and female *Cyp2c70*^−/−^ mice and WT littermates after 12 weeks of WTD feeding. E: Fecal bile acid excretion in male and female *Cyp2c70*^−/−^ mice and WT littermates after 12 weeks of WTD feeding. F: Hepatic mRNA levels of genes involved in bile acid synthesis and transport in male and female *Cyp2c70*^−/−^ mice and WT littermates after 12 weeks of WTD feeding. G: *Ileal* mRNA levels of genes involved in bile acid synthesis in male and female *Cyp2c70*^−/−^ mice and WT littermates after 12 weeks of WTD feeding. N = 7–10 mice/group. Data are presented as Tukey's box-and-whisker plots, and *P* values represent ∗*P* < 0.05, ∗∗*P* < 0.01, ∗∗∗*P* < 0.001 by Kruskal-Wallis H testing followed by Conover posthoc comparisons. *Bsep*, bile salt export pump; *Cyp7a1*, cholesterol 7α-hydroxylase; *Cyp8b1*, sterol 12α-hydroxylase; *Fgf15*, fibroblast growth factor 15; *Fxr*, farnesoid X receptor; *Ibabp*, ileal bile acid-binding protein; *Ntcp*, Na^+^-taurocholate cotransporting polypeptide; *Shp*, small heterodimeric partner.

We next explored concentrations and composition of BAs in WTD-challenged *Cyp2c70*^−/−^ mice. In line with the critical role of *Cyp2c70* in the generation of MCAs (11, 28), its absence was associated with a complete lack of these BA species and high abundances of CDCA in bile of WTD-fed *Cyp2c70*^−/−^ mice (Fig. 2A and supplemental Table S4). In addition, the proportion of 12α-hydroxylated BAs (CA and DCA) was markedly reduced, most prominently in female *Cyp2c70*^−/−^ mice (Fig. 2B). Accordingly, *Cyp2c70* deficiency induced a more hydrophobic BA pool in both male and female mice (Fig. 2C) with a hydrophobicity index comparable to that of humans (29). Corresponding differences in BA composition between *Cyp2c70*^−/−^ mice and their respective controls were observed in plasma (supplemental Tables S2 and S3 and supplemental Fig. S2A). Plasma total BA levels were moderately increased in male and female *Cyp2c70*^−/−^ mice compared with WT controls (Fig. 2D). The ratio unconjugated to conjugated BAs in plasma did not differ between *Cyp2c70*^−/−^ mice and their WT littermates (supplemental Fig. S2B). Fecal BA excretion, reflecting hepatic BA synthesis under steady-state conditions, was substantially reduced in both genders of WTD-fed *Cyp2c70*^−/−^ mice (Fig. 2E). Accordingly, hepatic mRNA expression of the BA synthesis enzymes *Cyp7a1* and *Cyp8b1* was lower in mice lacking *Cyp2c70* (Fig. 2F), the latter particularly in females. However, expression of *Nr1h4* (*Fxr*) was slightly decreased in livers of female *Cyp2c70*^−/−^ mice, and expression of its target genes *Nr0b2* (*Shp*) and *Abcb11* (*Bsep*) was not changed in livers of *Cyp2c70*^−/−^ mice compared with WT controls. Downregulation of the hepatic BA uptake transporter *Slc10a1* (*Ntcp*) (Fig. 2F) may contribute to the higher plasma BA levels in *Cyp2c70*^−/−^ mice. In the terminal ileum, *Shp* tended to be increased in female *Cyp2c70*^−/−^ mice but *Fabp6* (*Ibabp*) was upregulated in male *Cyp2c70*^−/−^ mice only compared with WT (Fig. 2G). However, ileal expression of *Fgf15*, which plays an important role in the regulation of BA synthesis, remained unchanged in *Cyp2c70*^−/−^ mice of both sexes (Fig. 2G). Thus, the impact of *Cyp2c70* deficiency on BA composition was observed in both genders but was more pronounced in female *Cyp2c70*^−/−^ mice.

### Effects of *Cyp2c70* deficiency on microbiome composition in WTD-fed mice

**Fig. 3:**
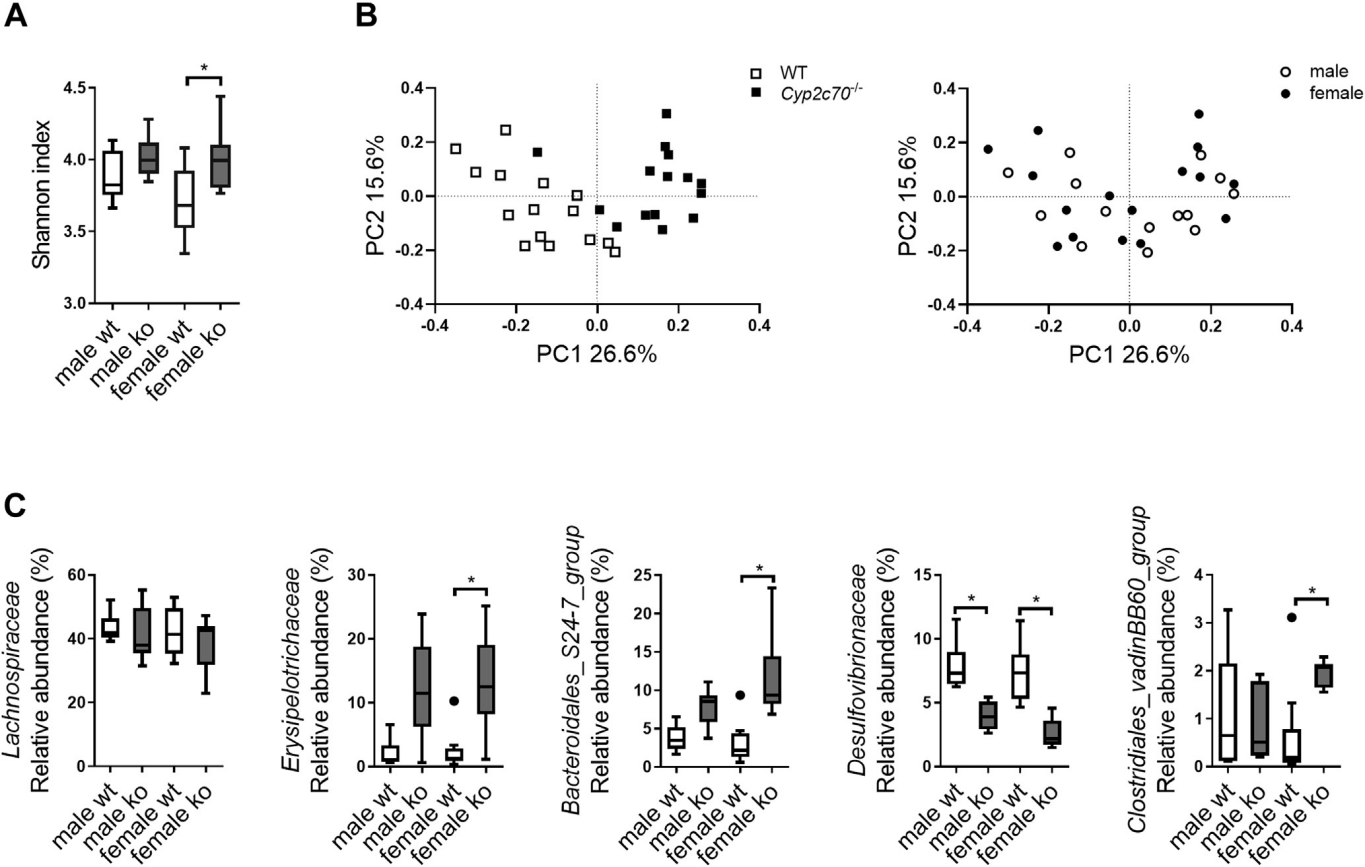
Effects of *Cyp2c70* deficiency on microbiome composition in WTD-fed mice. Bacterial DNA was extracted from cecum contents of mice fed a WTD for 12 weeks. 16s rRNA sequencing was performed with a total rarefaction of 119,545 reads per sample. Alpha diversity of the microbial composition was assessed using Shannon index (A), whereas PCoA (B) was performed using the Bray-Curtis distance of beta diversity. C: Relative abundance analysis of the gut microbiome between *Cyp2c70*^−/−^ and WT mice on the family level. The *P* values were corrected for multiple comparisons using the Benjamini-Hochberg method. N = 6–10 mice/group. Data are presented as Tukey's box-and-whisker plots. Mann-Whitney *U* nonparametric comparisons were used to compare *Cyp2c70*^−/−^ and WT mice within genders. ∗*P* < 0.05 for panel A, ∗adjusted *P* < 0.1 for panel C. PCoA, principal coordinates analysis; PC1, principal component 1; PC2, principal component 2.

BAs are known to modulate microbiome composition (30), and altered microbiome has been reported in human obesity (31) and implicated in the etiology of NAFLD (32). Therefore, we evaluated whether an MCA-deficient BA pool would modulate microbiome composition in mice fed an obesogenic diet. Figure 3A shows that alpha diversity of the cecal microbiome, as reflected by the Shannon index, did not differ between male *Cyp2c70*^−/−^ and WT mice but was higher in female *Cyp2c70*^−/−^ mice compared with controls. Principal coordinate analysis revealed that *Cyp2c70* deficiency rather than sex determined clustering (Fig. 3B), indicating that BA composition indeed is an important determinant of microbiome composition in WTD-fed mice. The analysis of relative abundances of gut microbiome at phylum level did not reveal marked differences between the groups (supplemental Fig. S3A). However, some remarkable differential abundances on the family level were observed between *Cyp2c70*^−/−^ and WT mice, especially in females (supplemental Fig. S3B). *Erysipelotrichaceae* and *Bacteroidales*_S24-7_group were more abundant in *Cyp2c70*^−/−^ mice compared with WT, whereas *Desulfovibrionaceae* showed a lower abundance (Fig. 3C). In addition, f_ Clostridiales_vadinBB60_group was only elevated in female *Cyp2c70*^*−*/−^ mice, whereas *Lachnospiraceae*, reported to be increased in patients with biliary fibrosis (33), did not differ between *Cyp2c70*^−/−^ and WT mice of either gender (Fig. 3C).

### A hydrophobic human-like BA composition prevents hepatic fat accumulation in WTD-challenged mice

**Fig. 4:**
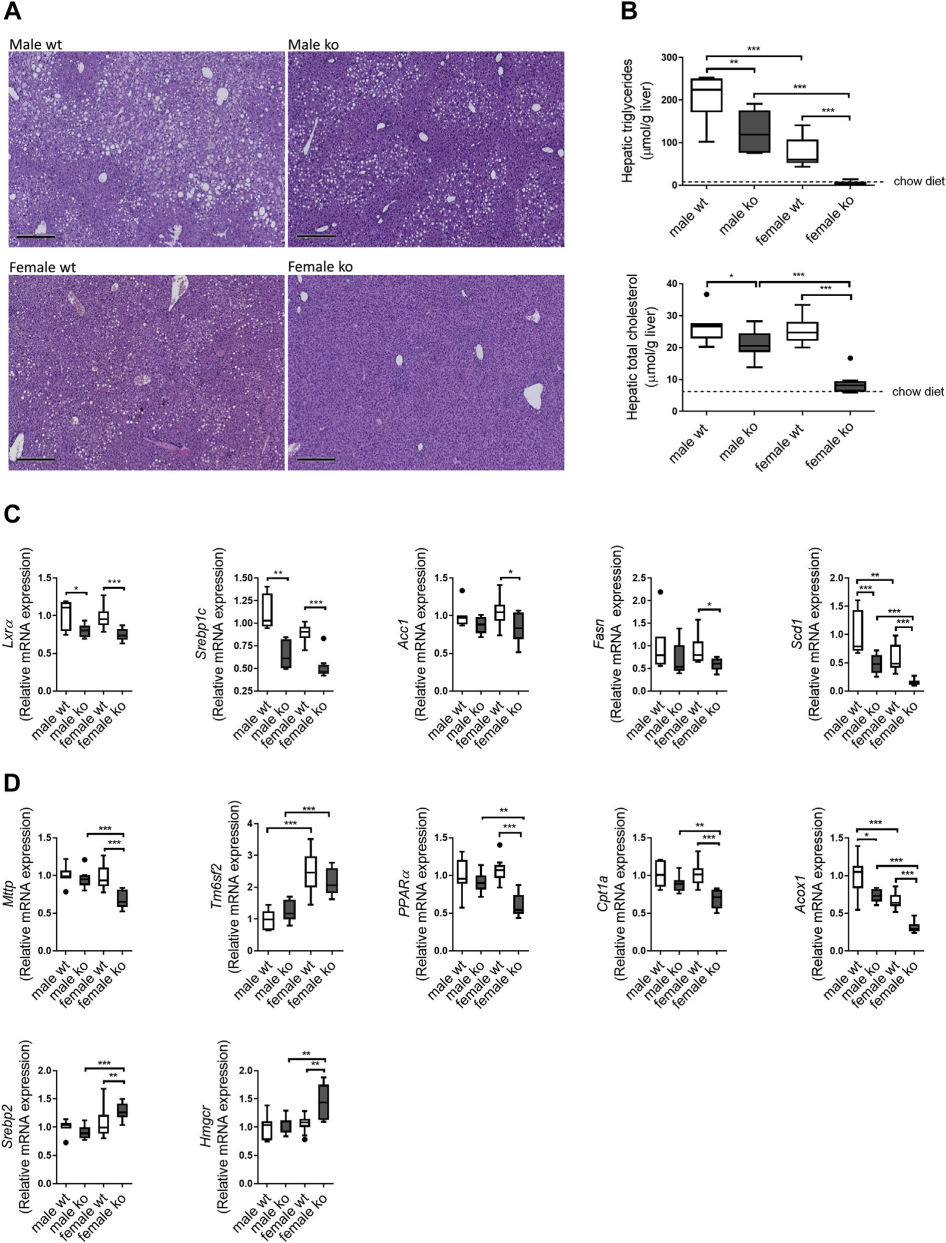
*Cyp2c70*^−/−^ mice are protected from WTD-induced hepatic steatosis. A: Representative images of H&E-stained liver sections of male and female *Cyp2c70*^−/−^ mice and WT littermates after 12 weeks of WTD feeding. Bar represents 300 μm. B: Hepatic triglyceride and cholesterol contents of male and female *Cyp2c70*^−/−^ mice and WT littermates after 12 weeks of WTD feeding, with average hepatic triglyceride and cholesterol contents in chow-fed female *Cyp2c70*^−/−^ mice indicated (dashed line) for comparison. C: Hepatic mRNA levels of *LXRα*, *Srebp1c*, and lipogenic genes (*Acc1*, *Fasn*, and *Scd1*) in male and female *Cyp2c70*^−/−^ mice and WT littermates after 12 weeks of WTD feeding. D: Hepatic mRNA levels of genes involved in VLDL assembly (*Mttp* and *Tm6sf2*), FA oxidation (*Pparα*, *Cpt1a*, and *Acox1*) and cholesterol synthesis (*Srebp2* and *Hmgcr*) in male and female *Cyp2c70*^−/−^ mice and WT littermates after 12 weeks of WTD feeding. N = 7–10 mice/group. Data are presented as Tukey's box-and-whisker plots. *P* values represent ∗*P* < 0.05, ∗∗*P* < 0.01, and ∗∗∗*P* < 0.001 by Kruskal-Wallis H testing followed by Conover posthoc comparisons. *Acc1*, acetyl-CoA carboxylase; *Acox1*, acyl-coenzyme A oxidase 1; *Cpt1a*, carnitine palmitoyltransferase 1A; *Fasn*, FA synthase; *Hmgcr*, 3-hydroxy-3-methyl-glutaryl-coenzyme A reductase; *Lxrα*, liver X receptor alpha; *Mttp*, microsomal triglyceride transfer protein; *Pparα*, peroxisome proliferator-activated receptor-alpha; *Scd1*, acyl-CoA desaturase 1; *Srebp1c*, sterol regulatory element-binding transcription protein 1c; *Srebp2*, sterol regulatory element-binding transcription protein 2; *Tm6sf2*, transmembrane 6 superfamily member 2.

H&E staining of liver sections revealed a clear presence of microvesicular and macrovesicular steatosis in pericentral areas in WT males and, to a lesser extent, in WT females (Fig. 4A). Lipid accumulation in male *Cyp2c70*^−/−^ mice was reduced compared with male WT, whereas, surprisingly, no steatosis could be detected in *Cyp2c70*^−/−^ females, which was confirmed by biochemical quantification (Fig. 4B). Importantly, hepatic lipid concentrations in WTD-fed female *Cyp2c70*^−/−^ mice were comparable to those found in chow-fed healthy young-adult C57BL/6J mice (12). NR1H3 (LXR_α_) is an oxysterol-activated nuclear receptor that regulates cholesterol homeostasis and lipogenesis. In association with reduced hepatic lipid contents in *Cyp2c70*^−/−^ mice, expression of *Lxrα* and *Srebp1c* as well as the genes encoding the enzymes involved in lipogenesis, *Acaca* (*Acc1*), *Fasn*, and *Scd1*, was reduced compared with WT mice (Fig. 4C). Hepatic mRNA expression of *Mttp* and *Tm6sf2*, both involved in VLDL assembly and secretion, and of genes involved in FA oxidation, that is, *Pparα, Cpt1a*, and *Acox1*, was not consistently affected in *Cyp2c70*^−/−^ mice (Fig. 4D). Furthermore, mRNA levels of *Srebp2* and its target gene *Hmgcr* were elevated in female *Cyp2c70*^−/−^ mice compared with WT (Fig. 4D).

Despite the marked differences in hepatic lipid accumulation between WTD-fed *Cyp2c70*^−/−^ mice and controls, plasma triglyceride levels were similar in all groups (supplemental Fig. S4A). Plasma nonesterified FAs and total cholesterol levels were significantly higher in male mice as compared with females, but no effect of *Cyp2c70* deficiency was observed (supplemental Fig. S4A). Compared with WT mice, female *Cyp2c70*^−/−^ mice had 2-fold higher plasma LDL-cholesterol levels (supplemental Fig. S4B). Hepatic mRNA expression of *Ldlr* was not significantly affected in female *Cyp2c70*^−/−^ mice compared with controls, but expression of *Pcsk9*, which induces LDLR degradation, was 2-fold higher in these animals (supplemental Fig. S4C), suggesting that decreased hepatic uptake of LDL may underlie the increased plasma LDL-cholesterol levels in female *Cyp2c70*^−/−^ mice.

### Cholangiopathy is present in WTD-fed *Cyp2c70*^−/−^ mice

**Fig. 5:**
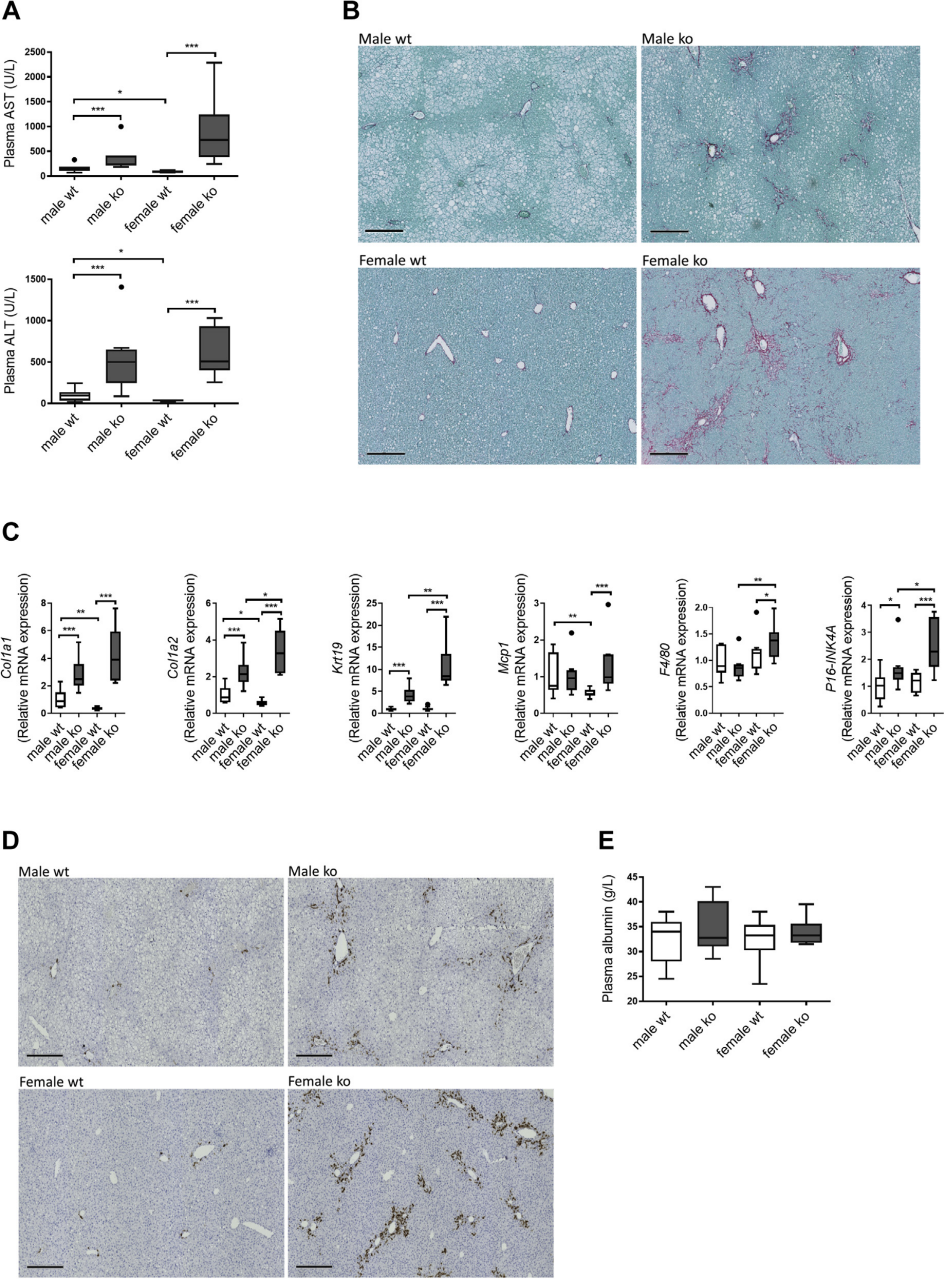
Cholangiopathy and portal fibrosis in WTD-fed *Cyp2c70*^−/−^ mice, particularly in females. A: Liver damage markers aminotransferase and alanine aminotransferase in plasma of male and female *Cyp2c70*^−/−^ mice and WT littermates after 12 weeks of WTD feeding. B: Representative images of Sirius Red–stained liver sections of male and female *Cyp2c70*^−/−^ mice and WT littermates after 12 weeks of WTD feeding. Bar represents 300 μm. C: Hepatic mRNA levels of genes involved in fibrogenesis, inflammation, and senescence in male and female *Cyp2c70*^−/−^ mice and WT littermates after 12 weeks of WTD feeding. D: Representative images of liver sections stained for the cholangiocyte marker CK19 in male and female *Cyp2c70*^−/−^ mice and WT littermates after 12 weeks of WTD feeding. Bar represents 300 μm. E: Plasma albumin concentrations in male and female *Cyp2c70*^−/−^ mice and WT littermates after 12 weeks of WTD feeding. N = 7–10 mice/group. Data are presented as Tukey's box-and-whisker plots. *P* values represent ∗*P* < 0.05, ∗∗*P* < 0.01, and ∗∗∗*P* < 0.001 by Kruskal-Wallis H testing followed by Conover posthoc comparisons. ALT, alanine aminotransferase; AST, aspartate aminotransferase; *Col1a1*, collagen type I alpha 1 chain; *Col1a2*, collagen type I alpha 2 chain; CK19, cytokeratin 19; *Krt19*, keratin 19; *Mcp1*, monocyte-chemoattractant protein 1; *Emr1*, *F4/80*, EGF-like module-containing mucin-like hormone receptor-like 1; *Cdkn2a*, *P16-INK4A*, cyclin-dependent kinase inhibitor 2A.

Plasma transaminases were significantly elevated in WTD-fed male and female *Cyp2c70*^−/−^ mice (Fig. 5A), as reported previously in chow-fed animals (12). In addition, Sirius Red staining showed collagen deposition in portal areas in *Cyp2c70*^−/−^ mice, which was more prominent in female *Cyp2c70*^−/−^ mice (Fig. 5B). Expression of fibrotic markers such as *Col1a1* and *Col1a2* was induced in livers of *Cyp2c70*^−/−^ mice of both sexes (Fig. 5C). However, we only observed a slightly increased expression of *Ccl2* (*Mcp1*), *F4/80*, and *Icam1*, but not of *Cd68*, *Tnfα*, *IL-1α*, *IL-1β*, *IL-10*, and *INFγ* in female *Cyp2c70*^−/−^ mice, whereas all inflammatory markers measured in this study remained unchanged in male *Cyp2c70*^−/−^ mice compared with controls (Fig. 5C and supplemental Fig. S5A). Hepatic expression of *Krt19*, encoding the cholangiocyte marker CK19, was higher in *Cyp2c70*^−/−^ mice (Fig. 5C). CK19 immunostaining confirmed the presence of increased numbers of cholangiocytes in portal areas of the livers from *Cyp2c70*^−/−^ mice, which was more pronounced in females (Fig. 5D) as reported previously in chow-fed conditions (12). Expression of *P16-INK4A*, a marker for senescence, was elevated in the liver of both male and female *Cyp2c70*^−/−^ mice compared with controls (Fig. 5C), suggesting that senescence might be involved in the development of cholangiopathy in *Cyp2c70*^−/−^ mice. However, expression of other senescence markers, *P21* and *P53*, remained unchanged (supplemental Fig. S5B). Despite the evident fibrosis in *Cyp2c70*^−/−^ mice, plasma albumin levels were comparable among all four groups (Fig. 5E), indicating that liver function is maintained in WTD-fed *Cyp2c70*^−/−^ mice.

### Reduced fat absorption rather than activation of BAT prevents steatosis in WTD-fed *Cyp2c70*^−/−^ mice

**Fig. 6:**
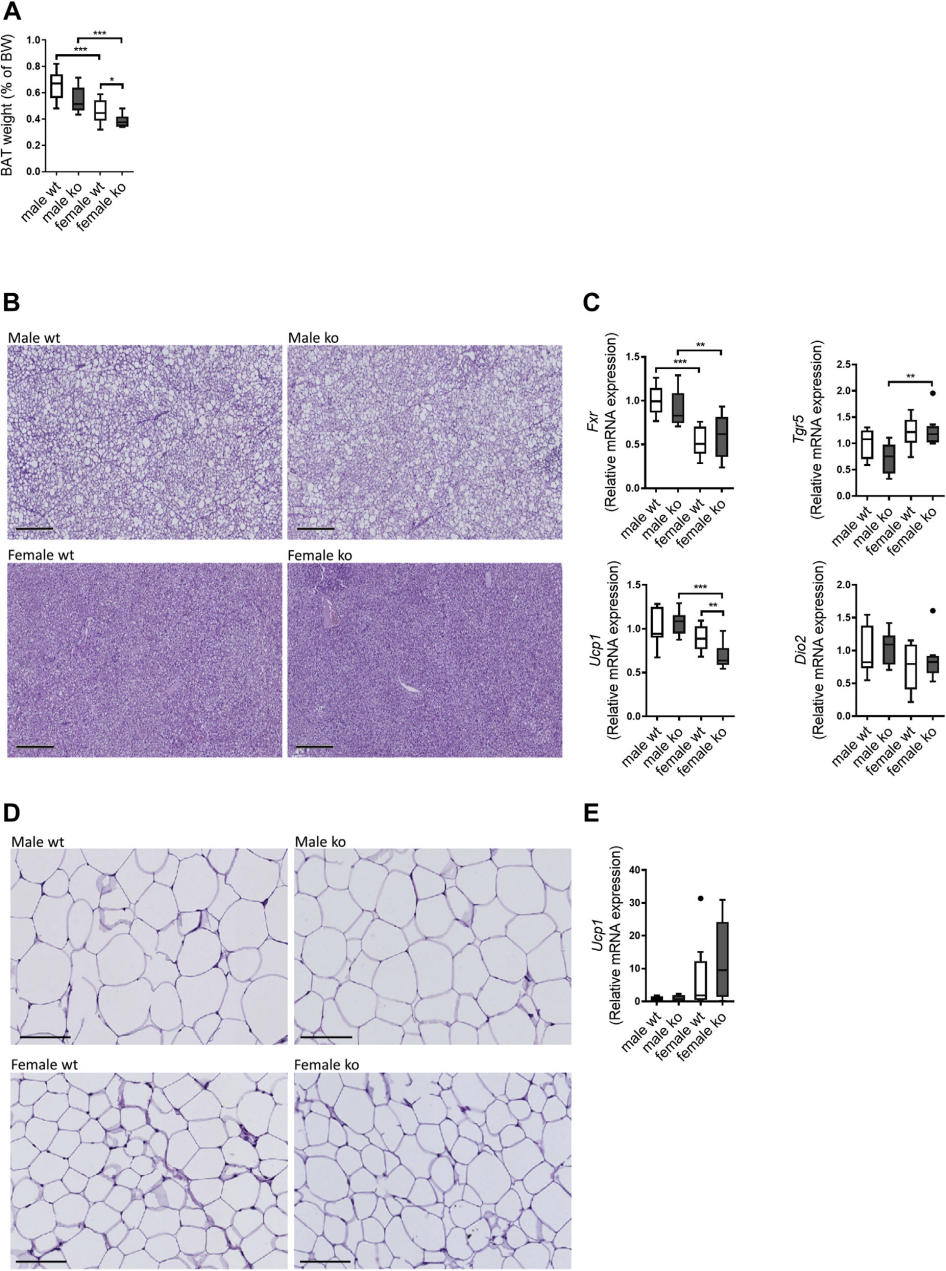
No indications for stimulation of BAT or browning of white adipose depots in WTD-fed *Cyp2c70*^−/−^ mice. A: BAT weights as percent of BW in male and female *Cyp2c70*^−/−^ mice and WT littermates after 12 weeks of WTD feeding. B: Representative images of H&E-stained BAT from male and female *Cyp2c70*^−/−^ mice and WT littermates after 12 weeks of WTD feeding. Bar represents 300 μm. C: BAT mRNA levels of bile acids receptors *Fxr* and *Tgr5* and thermogenic genes *Ucp1* and *Dio2* in male and female *Cyp2c70*^−/−^ mice and WT littermates after 12 weeks of WTD feeding. D: Representative images of H&E-stained subcutaneous white adipose tissue from male and female *Cyp2c70*^−/−^ mice and WT littermates after 12 weeks of WTD feeding. Bar represents 100 μm. E: mRNA levels of the thermogenic gene *Ucp1* in subcutaneous white adipose tissue in male and female *Cyp2c70*^−/−^ mice and WT littermates after 12 weeks of WTD feeding. N = 7–10 mice/group. Data are presented as Tukey's box-and-whisker plots. *P* values represent ∗*P* < 0.05, ∗∗*P* < 0.01, and ∗∗∗*P* < 0.001 by Kruskal-Wallis H testing followed by Conover posthoc comparisons. *Dio2*, iodothyronine deiodinase 2; *Fxr*, farnesoid X receptor; *Tgr5*, takeda G protein-coupled receptor 5; *Ucp1*, mitochondrial uncoupling protein 1.

We hypothesized that the hydrophobic BA pool might activate TGR5 in BAT and hence stimulate thermogenesis. BAT weights were not changed between male *Cyp2c70*^−/−^ and controls and were even slightly reduced in female *Cyp2c70*^−/−^ mice (Fig. 6A). Histology showed more lipid accumulation in BAT of male mice compared with females, but no differences were observed between genotypes (Fig. 6B). Furthermore, expression of *Fxr* and *Tgr5* remained unaffected by *Cyp2c70* deficiency, and no increase was observed in expression of *Ucp1* and *Dio2*, encoding key players in thermogenesis (Fig. 6C). Browning of scWAT can also contribute to increased thermogenesis. Compared with males, female mice had smaller adipocytes in scWAT, but no differences were observed between genotypes (Fig. 6D). Expression of *Ucp1* in scWAT was also similar between genotypes (Fig. 6E), indicating that there were no differences in browning of WAT between *Cyp2c70*^−/−^ mice and WT mice. It is, therefore, unlikely that activation of BAT or browning of WAT are primary reasons for the decreased adiposity and liver fat accumulation in WTD-fed *Cyp2c70*^−/−^ mice.

**Fig. 7:**
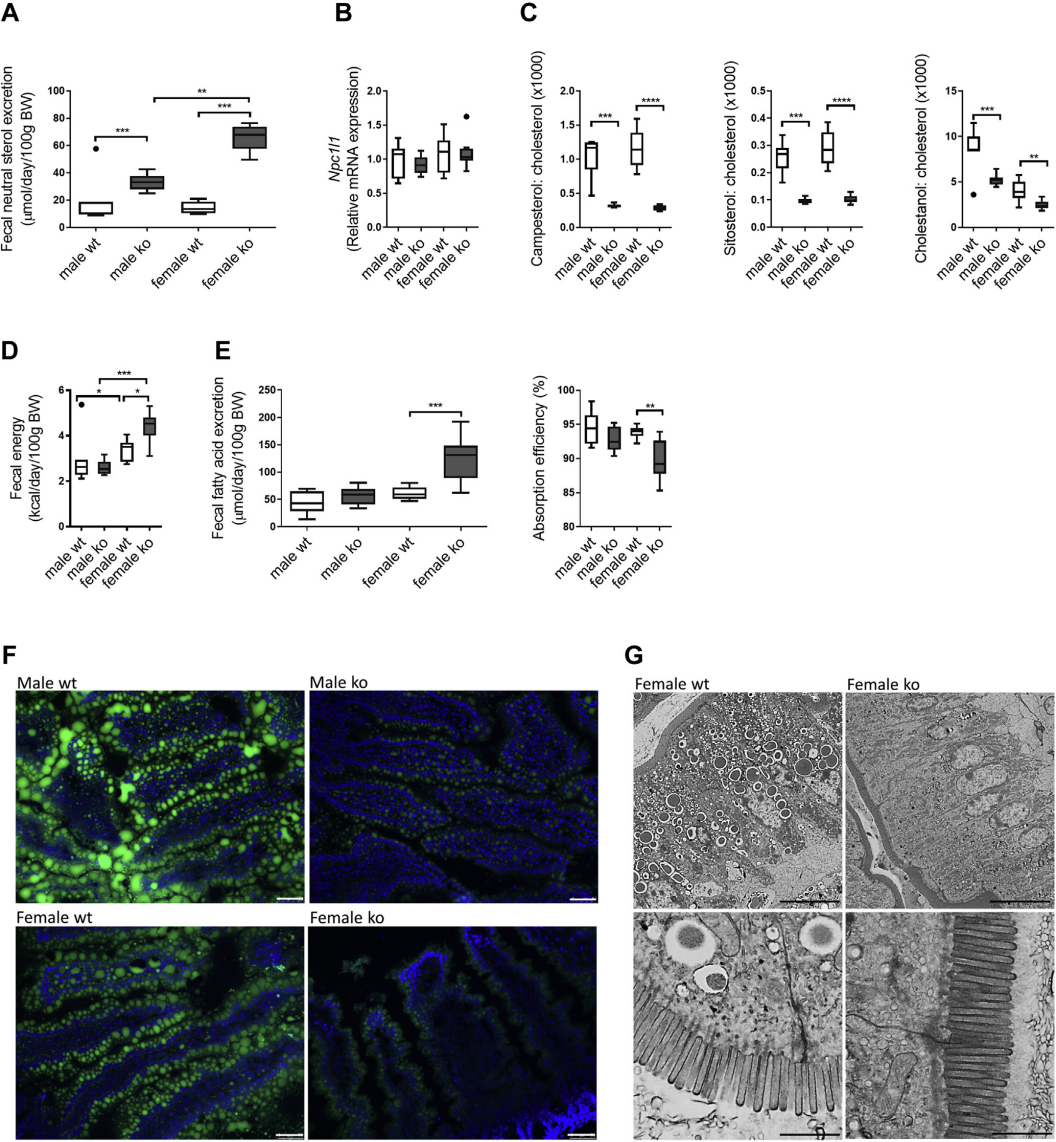
Cholesterol and FA absorption are reduced in WTD-fed *Cyp2c70*^−/−^ mice, particularly in females. A: Fecal neutral sterol excretion in male and female *Cyp2c70*^−/−^ mice and WT littermates after 12 weeks of WTD feeding. B: mRNA levels of the cholesterol transporter *Npc1l1* in jejunum of male and female *Cyp2c70*^−/−^ mice and WT littermates after 12 weeks of WTD feeding. C: Plasma concentrations of biomarkers of cholesterol absorption (campesterol, sitosterol, and cholestanol) normalized to cholesterol in male and female *Cyp2c70*^−/−^ mice and WT littermates after 12 weeks of WTD feeding. D: Fecal energy content in male and female *Cyp2c70*^−/−^ mice and WT littermates after 12 weeks of WTD feeding determined by bomb calorimetry. E: Total fecal FA excretion (left panel) and fat absorption efficiency (right panel) in male and female *Cyp2c70*^−/−^ mice and WT littermates after 12 weeks of WTD feeding. Fecal FA composition can be found in supplemental Table S5. F: Representative fluorescence microscope images of jejunum collected 2 h after gavage with BODIPY-labeled palmitic acid in olive oil in male and female *Cyp2c70*^−/−^ mice and WT littermates after 8 weeks of WTD feeding. Bar represents 50 μm. G: Representative transmission electron micrographs of jejunum collected from 8-week WTD-fed female *Cyp2c70*^−/−^ mice and WT littermates at 2 h after BODIPY-labeled oil gavage. Bar represents 5 μm for upper panels and 1 μm for lower panels. N = 6–10 mice/group. Data are presented as Tukey's box-and-whisker plots. *P* values represent ∗*P* < 0.05, ∗∗*P* < 0.01, and ∗∗∗*P* < 0.001 by Kruskal-Wallis H testing followed by Conover posthoc comparisons. *Npc1l1*, Niemann-Pick C1-like intracellular cholesterol transporter 1.

Next, we assessed the impact of the hydrophobic BA pool in *Cyp2c70*^−/−^ mice on the intestinal absorption of cholesterol and FAs. Fecal excretion of neutral sterols, that is, cholesterol and its bacterial derivatives, was strongly increased in male and female *Cyp2c70*^−/−^ mice compared with WT controls (Fig. 7A), whereas expression of the intestinal cholesterol importer *Npc1l1* was not altered (Fig. 7B). Yet, plasma non-cholesterol sterols that serve as surrogate markers of cholesterol absorption were markedly lower in *Cyp2c70*^−/−^ mice of both sexes (Fig. 7C). Only female *Cyp2c70*^−/−^ mice lost more energy in feces compared with WT controls (Fig. 7D). To obtain more insight in the efficiency of intestinal fat absorption, we quantified fecal FA excretion. Only female *Cyp2c70*^−/−^ mice excreted more FAs compared with controls. Especially the fecal contents of very long-chain FAs, which are highly dependent on BAs for their absorption, were increased (Fig. 7E and supplemental Table S5). Fat absorption efficiency, calculated as the difference between daily intake and daily fecal loss, was slightly but significantly reduced from 93% to 88% in female *Cyp2c70*^−/−^ mice compared with WT (Fig. 7E). To evaluate whether fat uptake by enterocytes or its subsequent transport by chylomicrons was primarily affected, mice on WTD for 8 weeks were fasted overnight and then gavaged with BODIPY-labeled palmitic acid in olive oil. Fluorescence microscopy of jejunal sections revealed that intestinal FA uptake was indeed reduced in *Cyp2c70*^−/−^ mice. Surprisingly, reduction of FA uptake by the enterocytes appeared to be similar in male and female *Cyp2c70*-deficient mice (Fig. 7F). Impaired enterocytic fat uptake was confirmed by transmission electron microscopy (TEM) (Fig. 7G), which clearly showed fewer lipid droplets within enterocytes of female *Cyp2c70*^−/−^ mice compared with WT. Importantly, TEM also revealed that the structure of the intestinal microvilli was not affected in *Cyp2c70*^−/−^ mice (Fig. 7G).

### Reduction of 12α-hydroxylated BAs contributes to reduced fat absorption in *Cyp2c70*^−/−^ mice

**Fig. 8:**
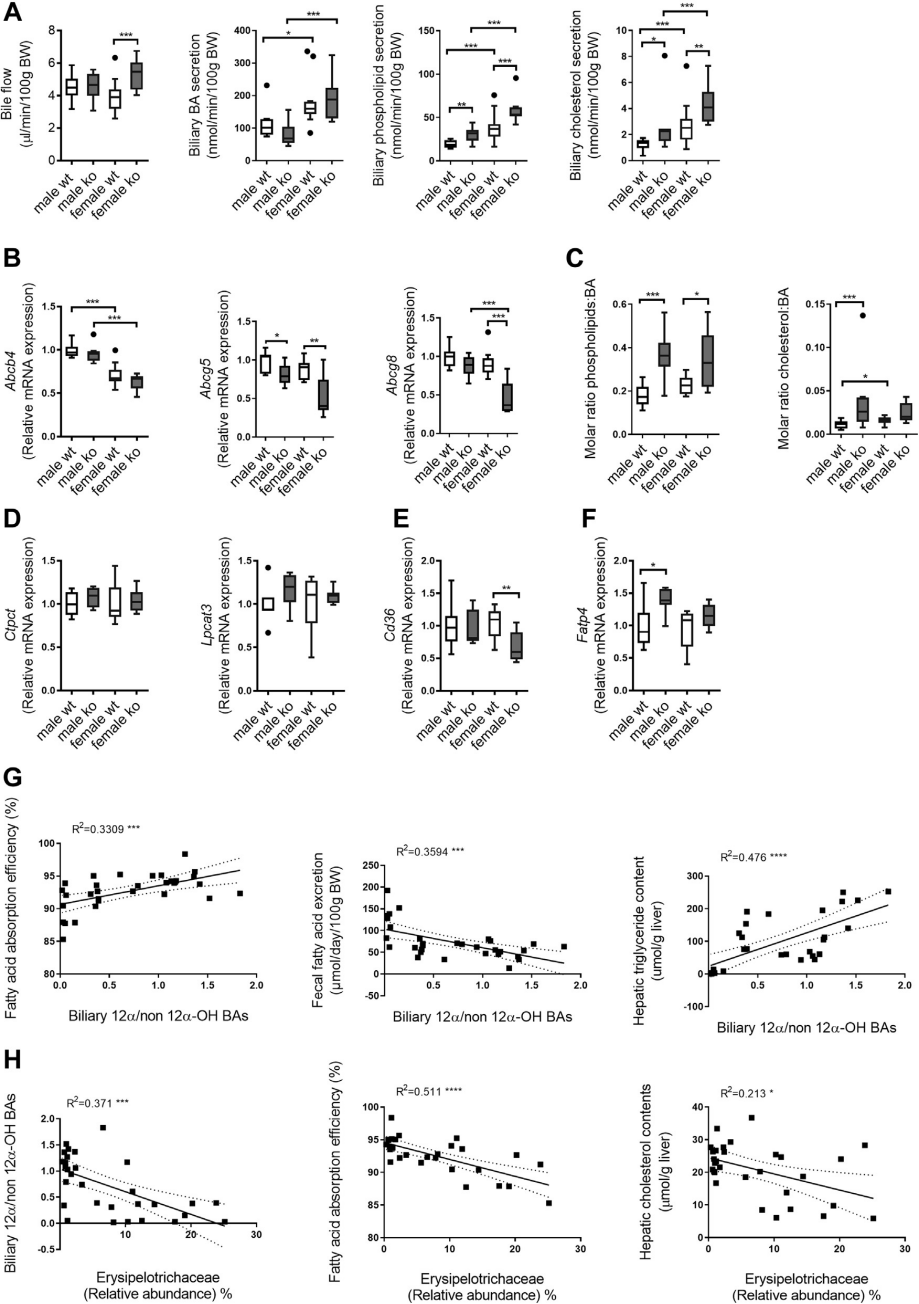
Altered bile acid composition rather than perturbed bile formation explains impaired fat absorption in WTD-fed *Cyp2c70*^−/−^ mice. A: Bile flow and biliary secretion rates of bile acids, phospholipids, and cholesterol in male and female *Cyp2c70*^−/−^ mice and WT littermates after 8 weeks of WTD feeding. B: Hepatic mRNA levels of genes involved in biliary phospholipid and cholesterol secretion in male and female *Cyp2c70*^−/−^ mice and WT littermates after 12 weeks of WTD feeding. C: Molar ratios of phospholipids and cholesterol, respectively, to bile acids in bile of male and female *Cyp2c70*^−/−^ mice and WT littermates after 8 weeks of WTD feeding. D: mRNA levels of genes involved in enterocytic phosphatidylcholine synthesis, in jejunum of male and female *Cyp2c70*^−/−^ mice and WT littermates after 12 weeks of WTD feeding. E: mRNA expression of *Cd36*, an FA transporter, in jejunum of male and female *Cyp2c70*^−/−^ mice and WT littermates after 12 weeks of WTD feeding. F: mRNA levels of *Fatp4*, involved in enterocytic triglyceride transport in jejunum of male and female *Cyp2c70*^−/−^ mice and WT littermates after 12 weeks of WTD feeding. G: Relationships between biliary 12α/non-12α-hydroxylated bile acids and FA absorption (left panel), fecal FA excretion (middle panel), and hepatic triglyceride content (right panel) across all groups of male and female *Cyp2c70*^−/−^ mice and WT littermates after 12 weeks of WTD feeding. H: The associations between the relative abundances of *Erysipelotrichaceae* (%) and biliary 12α/non-12α hydroxylated BAs (left panel), FA absorption (middle panel), and hepatic total cholesterol levels (right panel). N = 7–10 mice/group. Significance of differences between groups was accessed using Kruskal-Wallis H testing followed by Conover posthoc comparisons (panels A–F), whereas linear correlation analysis was performed in panels G–H. ∗*P* < 0.05, ∗∗*P* < 0.01, ∗∗∗*P* < 0.001, and ∗∗∗∗*P* < 0.0001. *Abcb4*, ATP-binding cassette subfamily B member 4; *Abcg5*, ATP-binding cassette subfamily G member 5; *Abcg8*, ATP-binding cassette subfamily G member 8; *Cd36*, cluster of differentiation 36; *Ctpct*, cytidine 5′-triphosphate: phosphocholine cytidylyltransferase; *Fatp4*, FA transport protein 4; *Lpcat3*, lysophosphatidylcholine acyltransferase 3.

Since both the quantity and the physicochemical properties of BAs in the intestine (6) as well as the biliary PL content (34) may control intestinal lipid absorption, we assessed bile formation in WTD-fed male and female *Cyp2c70*^−/−^ mice and their WT controls. Bile flow was not decreased in *Cyp2c70*^−/−^ mice compared with WT (Fig. 8A), indicating the absence of cholestasis in WTD-fed *Cyp2c70*^−/−^ mice. Biliary total BA secretion was unaffected by *Cyp2c70* deficiency (Fig. 8A). However, despite unaltered expression of the hepatic PL transporter *Abcb4* and reduced expression of the sterol transporter *Abcg5/8* (Fig. 8B), biliary PL and cholesterol secretion was stimulated in both male and female *Cyp2c70*^−/−^ mice (Fig. 8A). Hence, molar ratios of PL:BA and cholesterol:BA either were increased or tended to increase in *Cyp2c70*^−/−^ mice (Fig. 8C).

Because enterocytic PL metabolism impacts intestinal lipid absorption (18, 35), we measured expression levels of key genes involved in PL synthesis and remodeling in the jejunum. Expression of *Pcyt1a* (*Ctpct*) and *Lpcat3* was, however, unaltered (Fig. 8D). Gene expression of *Cd36*, a major long-chain FA transporter, was unaffected in males but significantly downregulated in the jejunum of female *Cyp2c70*^−/−^ mice (Fig. 8E); however, no differences in CD36 protein levels were detectable by Western blotting (*data not shown*). Another suggested transporter of long-chain FAs, *Fatp4*, was upregulated in the jejunum of male but not of female *Cyp2c70*^−/−^ mice (Fig. 8F).

Since altered PL metabolism or impaired expression of FA transporters do not seem to be affected in *Cyp2c70*^−/−^ mice, the altered BA composition represents the most conceivable cause for the impaired FA uptake and overall slightly reduced intestinal lipid absorption in *Cyp2c70*^−/−^ mice. Interestingly, we observed a positive correlation between the ratio of 12α/non-12α-hydroxylated BAs in bile and intestinal fat absorption efficiency and consequently, a negative correlation with fecal FA loss (Fig. 8G). In addition, this ratio correlated positively with hepatic triglyceride concentrations across the groups (Fig. 8G), indicating a crucial role of 12α-hydroxylated BA in development of NAFLD. Furthermore, the proportion of 12α-hydroxylated BAs appeared to be associated with gut microbiome composition. The relative abundance of *Erysipelotrichaceae* was negatively related to biliary 12α/non-12α-hydroxylated BAs and also with fat absorption efficiency and hepatic total cholesterol levels (Fig. 8H), indicating potential interactions between microbiome, BAs, and/or fat absorption.

## Discussion

In this study, we demonstrate a remarkable impact of a “human-like” BA composition on development of diet-induced obesity and NAFLD in mice, using recently generated *Cyp2c70*^−/−^ mice that lack the mouse-/rat-specific hydrophilic muricholates (12, 13, 14). Particularly, our results demonstrate that, despite the presence of a relatively hydrophobic BA pool, female *Cyp2c70*^−/−^ mice are protected from WTD-induced obesity, insulin resistance and hepatic steatosis, whereas male *Cyp2c70*^−/−^ mice show decreased hepatic fat accumulation but similar BW gain and insulin sensitivity than WT. Intriguingly, both male and female *Cyp2c70*^−/−^ mice showed impaired FA uptake by jejunal enterocytes, whereas only in females, a slight but significant 5% reduction of intestinal fat absorption efficiency was observed that correlated with the ratio of 12α/non-12α-hydroxylated BAs in bile. This ratio, known to be increased in humans with insulin resistance and type 2 diabetes (36), is lower in female *Cyp2c70*^−/−^ mice than in corresponding males. This sex-dependent difference is due to a strong suppression of the expression of *Cyp8b1*, the gene encoding sterol 12α-hydroxylase that determines the ratio in which the primary BAs CA and CDCA are synthesized by the liver (12). Consistent with expression of *Cyp8b1* in the liver, female and male *Cyp2c70*^−/−^ mice showed a 95% and 72% reduction, respectively, of the ratio 12α/non-12α-hydroxylated BAs in bile compared with their WT controls. The current findings indicate that 12α-hydroxylated BAs play a specific role in intestinal lipid absorption and, consequently, impact obesity and NAFLD development.

Of note, protective effects of BA composition on WTD-induced obesity, insulin sensitivity, and hepatic steatosis have also been described in *Cyp8b1*^−/−^ mice (37, 38, 39). However, the BA pool of *Cyp8b1*^−/−^ mice almost completely consists of MCAs, that is, BAs with high critical micellar concentrations (40) and FXR-antagonistic actions (10). Clearly, this hampers translation of these outcomes to the human situation. In contrast, the BA pool of *Cyp2c70*^−/−^ mice consists exclusively of BAs that are also present in humans, and the hydrophobicity index of biliary BAs in *Cyp2c70*^−/−^ mice is similar as the index in human bile (29). Recent work from our laboratory has demonstrated the existence of large interindividual variations in human BA pool composition in obese humans and, consequently, a very broad range (0.13–6.82) in the ratio of 12α/non-12α-hydroxylated BAs (8). In the current study, the ratio of 12α/non-12α-hydroxylated BAs in male *Cyp2c70*^−/−^ mice (0.31–0.61) fell in the lower part of the human range, whereas the ratio in female *Cyp2c70*^−/−^ mice was 0.02–0.06. The mechanisms for differential regulation of *Cyp8b1* in male and female *Cyp2c70*^−/−^ mice are not known. Hepatic mRNA levels of *Cyp8b1* are decreased by BA feeding of mice, stronger by CA and DCA than by CDCA, but CDCA will be converted to MCAs in WT mice (41). In addition, expression of *Cyp8b1* in mice is suppressed by cytokines and insulin (42, 43). However, hepatic cytokine expression was hardly affected in *Cyp2c70*^−/−^ mice, whereas plasma insulin levels were lower in female *Cyp2c70*^−/−^ mice compared with the corresponding parameters in male *Cyp2c70*^−/−^ mice. These parameters are therefore unlikely to contribute to the stronger reduction of hepatic *Cyp8b1* expression in female *Cyp2c70*^−/−^ mice compared with males. On the other hand, it has been reported that accumulation of hepatic cholesterol enhances (44), and the female hormone 17α-ethinylestradiol suppresses hepatic *Cyp8b1* expression (45). Therefore, less cholesterol accumulation in the liver, potentially leading to impaired LXRα signaling, and the presence of female hormones might (partly) explain the lower hepatic expression of *Cyp8b1* in female compared with male *Cyp2c70*^−/−^ mice. In addition, carbohydrate response element-binding protein activation by glucose-6-phosphate was shown to be involved in control of BA metabolism by suppressing *Cyp8b1* (46): whether altered intrahepatic glucose metabolism contributes to the strong downregulation of hepatic *Cyp8b1* expression that is observed in female *Cyp2c70*^−/−^ mice requires further investigation. In this respect, differences in zonation of *Cyp8b1* expression between male and female *Cyp2c70*^−/−^ mice might also be of great interest (47).

Despite the high abundance of CDCA, a potent endogenous FXR activator, in the BA pool of *Cyp2c70*^−/−^ mice, common FXR target genes were only marginally upregulated in ileum, whereas marked reductions of hepatic *Cyp7a1* and *Cyp8b1* expression were observed, in line with previous observations under chow-fed conditions (12). Consistent with our findings, Honda *et al.* (13) also reported no activation of FXR target genes in ileum or liver in *Cyp2c70*^−/−^ mice, and they suggested that hepatic inflammation, which they observed in male *Cyp2c70*^−/−^ mice only, triggered by hydrophobic BAs specifically downregulated the expression of *Cyp7a1* and *Cyp8b1*. Although hepatic inflammation was not severe in our *Cyp2c70*^−/−^ mice under WTD, we cannot rule out the possibility that inflammation might play a role in the downregulation of *Cyp7a1* and *Cyp8b1* expression in *Cyp2c70*^−/−^ mice.

Chow-fed adult *Cyp2c70*^−/−^ mice display biliary fibrosis and ductular reactions in their livers in an age-dependent manner, a feature that is more prominent in female *Cyp2c70*^−/−^ mice, and can be prevented by ursodeoxycholic acid treatment, indicating a role of BA hydrophobicity in this phenotype (12). Compared with chow-fed mice (12), 12 weeks of WTD did not evidently worsen biliary fibrosis in male or female *Cyp2c70*^−/−^ mice. Hepatic mRNA expression of inflammation markers, such as *Tnfα* and *Mcp1*, were reported to be positively correlated with hepatic unconjugated CDCA (13). When compared with chow-fed conditions (12), the elevated expression of the hepatic inflammation markers *Mcp1* and *Tnfα* in *Cyp2c70*^−/−^ mice compared with WT mice seems to be attenuated upon WTD feeding in both males and females. It is conceivable that WTD feeding induced a certain level of inflammation in livers of WT mice, which was not substantially enhanced by the hydrophobic BAs in *Cyp2c70*^−/−^ mice. In addition, elevated expression of *P16-INK4A* in *Cyp2c70*^−/−^ mice indicates a role of cellular senescence in the development of liver pathology in these mice, as recently proposed by Ogrodnik *et al.* (48). As expected, both male and female WT mice developed hepatic steatosis without fibrosis during 12 weeks of WTD feeding, whereas there was significantly less fat accumulation in the livers of male *Cyp2c70*^−/−^ mice and no steatosis at all in livers of female *Cyp2c70*^−/−^ mice. Several factors may have contributed to this unexpected finding and have been addressed in this study. First, hydrophobic BAs are in general stronger activators of BA receptors, such as FXR and TGR5 (49), both known to be involved in control of lipid metabolism and energy homeostasis (15). Therefore, inhibition of hepatic lipogenesis through FXR activation (50) and induction of thermogenesis through TGR5 activation (51) in BAT and/or WAT might be expected to occur in *Cyp2c70*^−/−^ mice. Expression of lipogenic genes was indeed reduced in livers of WTD-fed *Cyp2c70*^−/−^ mice of both sexes, and reduced lipogenesis may thus contribute to protection from WTD-induced steatosis, although it is generally accepted that the contribution of lipogenesis to NAFLD development is limited in mice (52). Furthermore, gene expression profiles in BAT and scWAT do not support a possible role for altered thermogenesis in WTD-fed *Cyp2c70*^−/−^ mice.

As an alternative mechanism, we have evaluated reduction of lipid absorption as a potential cause of protection from WTD-induced obesity and steatosis. It is known that fat absorption efficiency in high-fat diet-fed WT rodent models is very high and even remains >50% in complete absence of intestinal BAs (53). Somewhat counterintuitively, *Cyp2c70*^−/−^ mice of both sexes excreted more neutral sterols into feces and showed strongly reduced plasma levels of plant sterols, indicating impaired intestinal cholesterol absorption. Similar reductions of plasma plant sterol levels have been reported in chow-fed *Cyp2c70*^−/−^ mice (13). Expression of *Npc1l1*, encoding the major intestinal cholesterol transporter, was not affected in *Cyp2c70*^−/−^ mice, implying that other steps in enterocytic uptake and/or translocation processes must underlie the observed effects. Similarly, intestinal absorption of FAs appeared to be negatively affected by *Cyp2c70* deficiency, in this case particularly in females, as intestinal fat absorption efficiency was reduced from 93% to 88% in female *Cyp2c70*^−/−^ mice compared with WT. Intestinal fat absorption can be affected at multiple levels, that is, processes within the intestinal lumen, actual transport across the enterocytic apical membrane, and reconstitution of triglyceride molecules within the enterocytes, their packaging into chylomicrons, and secretion into lymph (54). Experiments with BODIPY-labeled palmitic acid demonstrated that FA entry into jejunal enterocytes was strongly impaired in both male and female *Cyp2c70*^−/−^ mice. This conclusion could be confirmed by TEM, showing an absence of lipid-laden vesicles in enterocytes of female *Cyp2c70*^−/−^ mice. The expression of putative FA transporters *Cd36* (55) and *Fatp4* (56) as well as genes encoding enzymes involved in enterocytic phosphatidylcholine synthesis (*Pcyta*) and remodeling (*Lpcat3*), recently shown to be crucial for enterocytic FA uptake (18, 35), was not or only minimally affected in female *Cyp2c70*^−/−^ mice. It thus appears that events within the intestinal lumen must be responsible. The fact that differences in intestinal lipid uptake were also observed after administration of FAs in their free form, that is, the palmitic acid bolus, indicated that the observed impaired intestinal fat absorption efficiency in *Cyp2c70*^−/−^ mice was, at least for a major part, independent of intestinal TG hydrolysis mediated by pancreatic enzymes. WTD-fed *Cyp2c70*^−/−^ mice showed unaffected bile flow and total biliary BA secretion rates, implying that a shortage of BAs in the intestinal lumen was not the reason for impaired fat absorption. In addition, bile produced by *Cyp2c70*^−/−^ mice contained more cholesterol and phospholipids, the latter being essential for fat absorption by providing surface material for chylomicron formation (34). Therefore, the most likely reason for impaired cholesterol and fat absorption appears to be the BA composition of *Cyp2c70*^−/−^ mice, with a high abundance of CDCA and, particularly in females, low amounts of CA. Indeed, fat absorption efficiency as well as hepatic cholesterol content, which is mainly diet derived under these conditions, correlated with the ratio 12α/non-12α-hydroxylated BAs in bile: low biliary CA is associated with low intestinal lipid absorption. Indeed, CA feeding has been reported to promote dietary fat and cholesterol absorption in WT mice (57) as well as in *Cyp7a1*^−/−^ mice (58) and *Cyp8b1*^−/−^ mice (59), possibly related to specific functions of taurine-conjugated CA in the mixed micelles in the intestinal lumen (60). It is important to keep in mind that the human and murine BA composition is not optimized for maximal solubilization of lipids but for maximal facilitation of lipid absorption. This is reached by a trade-off between efficient solubilization of lipids within the intestinal lumen and relatively high concentrations of lipids in the aqueous phase to allow interactions with the membranes and transporters of cells lining the intestinal lumen (61). CA and DCA appear to be important for the latter part. Indeed, muricholates are known to be poor detergents, and feeding of mice with either MCAs or CDCA, which will rapidly be converted to MCAs in mice, inhibits cholesterol absorption, whereas CA feeding strongly promotes this process (57). In the *Cyp2c70*^−/−^ mouse model, both deficiency of muricholates and enrichment of the pool with CDCA might enhance lipid absorption while, at the same time, the strong reduction of 12α-hydroxylated BAs (CA and DCA) will have the opposite effect. The overall impairment of lipid and cholesterol absorption observed in *Cyp2c70*^−/−^ mice indicates that the reduction in 12α-hydroxylated BAs actually overrules the potentially stimulatory effects of changes in MCAs and CDCA. This is supported by data from human studies, showing that CDCA treatment (20 days, 15 mg/kg/day) decreases average cholesterol absorption from ∼33% to ∼15%, coinciding with a reduction in the contribution of 12α-hydroxylated species to biliary BAs from 55% to 15% (62). Therefore, we propose that the very low production of these 12α-hydroxylated BAs in female *Cyp2c70*^−/−^ mice, because of the sex-dependent *Cyp8b1* suppression, reduces fat and cholesterol absorption efficiency and thereby prevents development of WTD-induced obesity and NAFLD. This phenotype is less pronounced in male *Cyp2c70*^−/−^ mice, likely because of the presence of a larger proportion of CA in their BA pools than in females. It is evident, however, that cholesterol absorption, a process very dependent on intestinal BAs (63), is also impaired in males, whereas FA absorption appears to be delayed, that is, impaired jejunal uptake can be compensated at more distal sites of the small intestine. Yet, lower hepatic fat content coexisting with unaffected adipocyte depots suggests that distribution of dietary fat is altered in male *Cyp2c70*^−/−^ mice.

Since BAs play an important role in shaping of the microbiome (30) and, in turn, microbiome composition has been related to NAFLD/NASH development in humans (32), we assessed cecal microbiome composition. It is evident that *Cyp2c70* deficiency, likely through its effect on biliary BA composition, is a major determinant of microbiome composition in mice. In the acute hepatic *Cyp2c70* knockdown mouse model, modulation of BA pool did not translate into alterations of fecal microbiota within 4 weeks (28), whereas in mice with lifelong full body *Cyp2c70* deficiency, the microbiome signature showed certain similarities with the alterations in gut flora observed in patients with primary biliary cholangitis (12). However, under WTD, it appeared that differences in gut microbiome profile between *Cyp2c70*^−/−^ mice and their WT controls were also related to diet composition and differences in fat absorption efficiency. Amelioration of WTD-induced obesity and hepatic steatosis in female *Cyp2c70*^−/−^ mice was associated with a “beneficial microbiome profile,” including lower abundance of *Desulfovibrionaceae*, which has been related to diet-induced obesity (64), and higher abundances of *Bacteroidales_S24-7_group* and *Erysipelotrichaceae*, which have been associated with improvement of diet-induced obesity (65). *Erysipelotrichaceae* has been reported to positively correlate with fecal fat excretion and negatively with fat digestibility in dogs (65). In our study, the relative abundance of *Erysipelotrichaceae* negatively correlated with biliary 12α/non-12α-hydroxylated BA ratios as well as with fat absorption efficiency. Interestingly, *Erysipelotrichaceae* also negatively correlated with total cholesterol content in the liver. The higher abundance of *Erysipelotrichaceae* may be a consequence of altered BA composition or reduced fat absorption in *Cyp2c70*^−/−^ mice. Yet, a causal role of specific bacterial species in the phenotype cannot be excluded.

It is evident that several issues still need to be addressed. In contrast to humans, mice rehydroxylate DCA upon its return to the liver to form CA again. Simultaneous deletion of both *Cyp2c70* and *Cyp2a12* overcomes this discrepancy, as shown by Honda *et al.* (13), as the potential roles of both 12α-hydroxylated BAs, that is, CA and DCA, in NAFLD development need further investigation. Also, differences in microbiome composition in the cecum may be a consequence of differences in BA composition or of the entry of excess FAs and sterols into the cecum. The current study neither does allow to dissect these potential mechanisms nor does allow to elucidate the contribution of altered microbiome in the observed prevention of NAFLD in *Cyp2c70*^−/−^ mice.

To conclude, our data demonstrate that amelioration of WTD-induced obesity and NAFLD development in *Cyp2c70*^−/−^ mice is mainly driven by a relatively small reduction of dietary lipid absorption in combination with reduced hepatic de novo lipogenesis. In this respect, 12α-hydroxylated BAs seem to be superior in facilitating FA and cholesterol absorption compared with non-12α-hydroxylated BAs. Given the great variability in the ratios at which CA and CDCA are being produced in the human liver, also in healthy subjects (8, 9), it will be of great interest to evaluate whether this heterogeneity contributes to the reported interindividual variations in cholesterol (66) and fat (67) absorption efficiency. In this respect, we have recently demonstrated that plasma levels of the 12α-hydroxylated secondary BA DCA are associated with hepatic fat content in obese subjects (8). Hence, further studies on the role of endogenous 12α-hydroxylated BAs in the development of NAFLD in humans are warranted.

## Data availability

Large-scale zoomable electron microscopy data at full resolution are available through www.nanotomy.org when the article is accepted. For review purposes, the data are now available at www.nanotomy.org/OA/Li2021SUB. All remaining data are contained within the article.

## Supplemental data

This article contains supplemental data.

## Conflict of interest

The authors declare that they have no conflicts of interest with the contents of this article.
