# Supplementary material for: Low production of 12α-hydroxylated bile acids prevents hepatic steatosis in Cyp2c70−/− mice by reducing fat absorption
Source: J Lipid Res. 2021 Oct 7;62:100134. doi: 10.1016/j.jlr.2021.100134 (PMC8596750; doi:10.1016/j.jlr.2021.100134)
Supplement: Supplemental Tables S1–S5 and Figures S1–S5 [file mmc1.docx]

**Supplemental Material**

**Low production of 12α-hydroxylated bile acids prevents hepatic steatosis in *Cyp2c70*^-/-^ mice by reducing fat absorption**

Rumei Li^1*^, Anna Palmiotti^1*^, Hilde D. de Vries^2^, Milaine V. Hovingh^1^, Martijn Koehorst ^2^, Niels L. Mulder^1^, Yue Zhang^1,3^, Kim Kats^4^, Vincent W. Bloks^1^, Jingyuan Fu^1,3^, Henkjan J. Verkade^1^, Jan Freark de Boer^1,2*^, Folkert Kuipers ^1,2*^

^1^Department of Pediatrics, University of Groningen, University Medical Center Groningen, Groningen, The Netherlands, ^2^Department of Laboratory Medicine, University of Groningen, University Medical Center Groningen, Groningen, The Netherlands, ^3^Department of Genetics, University of Groningen, University Medical Center Groningen, Groningen, The Netherlands, ^4^Department of Biomedical Science of Cells and Systems, University of Groningen, University Medical Center Groningen, Groningen, The Netherlands.

*equal contributions

**Supplemental Table S1: Glucose parameters**

|  |  | **Male** | | | **Female** | |
| --- | --- | --- | --- | --- | --- | --- |
|  |  | **WT**  **(n=7)** | ***Cyp2c70*^-/-^**  **(n=8)** | **WT**  **(n=10)** | | ***Cyp2c70*^-/-^**  **(n=8)** |
| *Chow diet* | |  |  |  | |  |
| Fasting glucose (mmol/L) | | 8.0 (7.8-8.9) | 8.1 (7.4-8.5) | 7.5 (7.0-9.0) | | 8.0 (6.8-8.3) |
| *After 11 weeks of WTD* | |  |  |  | |  |
| Fasting glucose (mmol/L) | | 10.7 (10.2-11.2) | 10.3 (9.8-11.6) | 9.6 (8.9-10.3) | | 7.9 (7.2-8.6)** |
| Fasting insulin (ng/mL) | | 1.1 (1.0-1.2) | 1.0 (0.9-1.3) | 0.5 (0.3-0.5) | | 0.2 (0.2-0.4)* |
| HOMA-IR | | 21.1 (18.4-24.6) | 19.6 (14.2-26.1) | 7.7 (6.0-9.3) | | 3.0 (2.3-4.8)* |

Data represents median ± interquartile range. P-values represent Mann-Whitney U nonparametric comparisons within genders. *p<0.05, **p<0.01. WT, wild-type; WTD, Western-type diet; HOMA-IR, homeostatic model assessment of insulin resistance.

**Supplemental Table S2. Plasma bile acid concentrations of *Cyp2c70*^-/-^ mice and their WT littermates after 12 weeks of WTD feeding.**

|  |  | **Male** | | | **Female** | |
| --- | --- | --- | --- | --- | --- | --- |
| **Concentration**  **(μmol/l)** | **WT**  **(n=7)** | | ***Cyp2c70*^-/-^**  **(n=8)** | **WT**  **(n=10)** | | ***Cyp2c70*^-/-^**  **(n=8)** |
| CA | 0.041  (0.026-0.135) | | 0.220  (0.131-0.280) | 0.089  (0.061-0.219) | | 0.192  (0.133-0.249) |
| TCA | 1.420  (0.363-2.500) | | 2.030  (1.361-3.148) | 1.610  (0.602-3.820) | | 0.765  (0.432-1.710) |
| DCA | 0.179  (0.146-0.195) | | 0.141  (0.135-0.166) | 0.274  (0.171-0.350) | | 0.058 ****  (0.054-0.067) |
| TDCA | 0.111  (0.091-0.254) | | 0.202  (0.164-0.292) | 0.446  (0.191-1.090) | | 0.086 **  (0.047-0.174) |
| CDCA | 0  (0-0.026) | | 0.865 **  (0.631-1.563) | 0  (0-0.032) | | 3.920 ****  (2.123-6.155) |
| TCDCA | 0.090  (0.033-0.111) | | 4.425 **  (3.080-9.873) | 0.110  (0.064-0.293) | | 21.250 **  (5.563-30.875) |
| αMCA | 0  (0-0) | | Undetected | 0  (0-0.046) | | Undetected |
| TαMCA | 0.111  (0.059-0.288) | | Undetected ** | 0.270  (0.141-0.675) | | Undetected **** |
| βMCA | 0.053  (0-0.083) | | Undetected * | 0.096  (0.080-0.142) | | Undetected **** |
| TβMCA | 0.184  (0.083-0.454) | | Undetected **** | 0.328  (0.157-0.787) | | Undetected **** |
| ωMCA | 0.069  (0.063-0.314) | | Undetected **** | 0.126  (0.071-0.206) | | Undetected ** |
| TωMCA | 0.095  (0.083-0.133) | | Undetected **** | 0.190  (0.074-0.253) | | Undetected ** |
| UDCA | 0.031  (0.029-0.036) | | 0.208  (0.137-0.260) | 0.040  (0.028-0.049) | | 1.153 **  (0.432-2.070) |
| TUDCA | 0.097  (0.073-0.150) | | 0.630 **  (0.365-0.923) | 0.234  (0.162-0.324) | | 2.590 **  (2.040-4.828) |
| LCA | 0.027  (0.013-0.034) | | 0.269 **  (0.192-0.313) | 0.037  (0.031-0.050) | | 0.922 **  (0.410-0.994) |
| TLCA | 0  (0-0) | | 0.126 **  (0.091-0.174) | 0  (0-0) | | 0.734 ****  (0.251-1.075) |
| HDCA | 0  (0-0) | | Undetected | 0  (0-0) | | 0  (0-0.054) |
| THDCA | 0  (0-0) | | 0  (0-0.013) | 0.071  (0-0.099) | | 0.133  (0.062-0.192) |
| Total | 2.470  (1.420-4.128) | | 9.546 *  (6.253-16.449) | 4.213  (1.967-8.315) | | 35.313 **  (18.661-43.061) |

Data represents median ± interquartile range. P-values represent Mann-Whitney U nonparametric comparisons within genders. *p<0.05, **p<0.01, ***p<0.001, ****p<0.0001. WT, wild-type; WTD, Western-type diet.

**Supplemental Table S3. Plasma bile acid compositions of *Cyp2c70*^-/-^ mice and their WT littermates after 12 weeks of WTD feeding.**

|  |  | **Male** | | | **Female** | |
| --- | --- | --- | --- | --- | --- | --- |
| **% of total BAs** | **WT**  **(n=7)** | | ***Cyp2c70*^-/-^**  **(n=8)** | **WT**  **(n=10)** | | ***Cyp2c70*^-/-^**  **(n=8)** |
| CA | 2.18%  (1.14%-9.05%) | | 1.80%  (1.31%-2.83%) | 3.33%  (1.05%-6.48%) | | 0.70%  (0.55%-0.95%) |
| TCA | 29.91%  (24.84%-57.87%) | | 18.23% **  (16.72%-20.41%) | 36.49%  (31.74%-45.86%) | | 2.35% ****  (1.36%-4.57%) |
| DCA | 6.15%  (3.98%-12.19%) | | 1.55% ***  (1.18%-2.26%) | 6.90%  (4.10%-8.67%) | | 0.17% ****  (0.15%-0.23%) |
| TDCA | 5.73%  (4.25%-7.24%) | | 2.56% *  (2.19%-2.87%) | 11.77%  (9.21%-13.18%) | | 0.27% ****  (0.14%-0.36%) |
| CDCA | 0  (0-0.78%) | | 8.05% ***  (6.42%-10.64%) | 0  (0-0.40%) | | 14.32% ****  (7.23%-22.67%) |
| TCDCA | 2.33%  (2.21%-2.76%) | | 54.72% ***  (46.58%-57.99%) | 3.10%  (2.77%-3.72%) | | 55.83% ****  (43.13%-70.34%) |
| αMCA | 0  (0-0) | | Undetected | 0  (0-1.07%) | | Undetected |
| TαMCA | 4.49%  (2.69%-5.88%) | | Undetected ** | 7.04%  (6.36%-7.71%) | | Undetected **** |
| βMCA | 1.28%  (0-6.17%) | | Undetected * | 3.57%  (1.52%-4.64%) | | Undetected **** |
| TβMCA | 7.45%  (5.74%-10.99%) | | Undetected *** | 7.87%  (7.05%-9.57%) | | Undetected **** |
| ωMCA | 2.80%  (1.86%-7.87%) | | Undetected ** | 3.42%  (1.95%-4.48%) | | Undetected **** |
| TωMCA | 3.38%  (3.28%-5.89%) | | Undetected *** | 3.36%  (3.03%-4.19%) | | Undetected **** |
| UDCA | 1.27%  (0.65%-2.38%) | | 1.67%  (1.16%-2.14%) | 0.72%  (0.45%-1.74%) | | 5.19%  (1.31%-7.05%) |
| TUDCA | 3.91%  (3.10%-4.83%) | | 6.57% *  (4.79%-7.35%) | 5.74%  (4.19%-8.25%) | | 12.33% *  (8.52%-14.00%) |
| LCA | 0.66%  (0.04%-2.18%) | | 2.55%  (1.91%-3.49%) | 0.99%  (0.64%-1.57%) | | 1.91%  (1.42%-2.88%) |
| TLCA | 0  (0-0) | | 1.61% ***  (0.99%-1.90%) | 0  (0-0) | | 1.77% ****  (1.57%-2.09%) |
| HDCA | 0  (0-0) | | Undetected | 0  (0-0) | | 0  (0-0.07%) |
| THDCA | 0  (0-0) | | 0  (0-0.08%) | 0.79%  (0-1.22%) | | 0.37%  (0.31%-0.46%) |

Data represents (median ± interquartile range) percentage of individual BA species in total plasma BAs. P-values represent Mann-Whitney U nonparametric comparisons within genders. *p<0.05, **p<0.01, ***p<0.001, ****p<0.0001. BAs. WT, wild-type; WTD, Western-type diet; BA, bile acid.

**Supplemental Table S4. Biliary bile acid compositions of *Cyp2c70*^-/-^ mice and their WT littermates after 12 weeks of WTD feeding.**

|  |  | **Male** | | | **Female** | |
| --- | --- | --- | --- | --- | --- | --- |
| **% of total BAs** | **WT**  **(n=6)** | | ***Cyp2c70*^-/-^**  **(n=8)** | **WT**  **(n=10)** | | ***Cyp2c70*^-/-^**  **(n=8)** |
| CA | 0.07%  (0.01%-0.22%) | | 0.08%  (0.05%-0.17%) | 0.14%  (0-0.17%) | | Undetected |
| TCA | 55.08%  (53.91%-58.03%) | | 25.24% ***  (24.44%-26.70%) | 46.86%  (46.01%-49.81%) | | 3.81% ***  (2.51%-4.85%) |
| DCA | Undetected | | Undetected | Undetected | | Undetected |
| TDCA | 1.79%  (1.68%-2.39%) | | 1.28% *  (1.02%-1.58%) | 3.66%  (2.99%-4.19%) | | 0.21% ****  (0.08%-0.33%) |
| CDCA | 0  (0-0) | | 0  (0-0) | Undetected | | 0  (0-0) |
| TCDCA | 3.22%  (3.06%-3.36%) | | 64.53% ***  (63.86%-67.37%) | 3.81%  (3.01%-4.36%) | | 80.22% ****  (77.57%-83.72%) |
| αMCA | 0  (0-0.04%) | | Undetected | 0.10%  (0.02%-0.13%) | | Undetected ** |
| TαMCA | 10.78%  (10.16%-11.33%) | | Undetected *** | 12.61%  (11.94%-13.22%) | | Undetected **** |
| βMCA | 0.17%  (0.11%-0.23%) | | Undetected *** | 0.09%  (0.08%-0.12%) | | Undetected **** |
| TβMCA | 20.90%  (20.28%-23.27%) | | Undetected *** | 25.22%  (23.77%-27.51%) | | Undetected **** |
| ωMCA | 0.01%  (0-0.05%) | | Undetected | 0.06%  (0.05%-0.09%) | | Undetected *** |
| TωMCA | 3.56%  (2.68%-3.92%) | | Undetected *** | 2.95%  (2.43%-3.69%) | | Undetected **** |
| UDCA | 0  (0-0) | | Undetected | Undetected | | Undetected |
| TUDCA | 2.71%  (2.53%-2.79%) | | 5.91% **  (4.68%-6.81%) | 3.46%  (2.91%-3.90%) | | 13.38% **  (9.55%-15.11%) |
| LCA | Undetected | | Undetected | Undetected | | Undetected |
| TLCA | 0  (0-0.01%) | | 0.64% ***  (0.50%-0.94%) | 0.05%  (0.05%-0.06%) | | 1.19% ****  (0.84%-1.47%) |
| HDCA | Undetected | | Undetected | Undetected | | Undetected |
| THDCA | 0.36%  (0.30%-0.51%) | | 0.23%  (0.21%-0.29%) | 0.58%  (0.45%-0.65%) | | 0.39%  (0.35%-0.44%) |

Data represents (median ± interquartile range) percentage of individual BA species in total biliary BAs. P-values represent Mann-Whitney U nonparametric comparisons within genders. *p<0.05, **p<0.01, ***p<0.001, ****p<0.0001. WT, wild-type; WTD, Western-type diet; BA, bile acid.

**Supplemental Table S5. Fecal fatty acid profiles of *Cyp2c70*^-/-^ mice and their WT littermates after 12 weeks of WTD feeding.**

|  |  | **Male** | | | **Female** | |
| --- | --- | --- | --- | --- | --- | --- |
| **μmol/day/100g BW** | **WT**  **(n=6)** | | ***Cyp2c70*^-/-^**  **(n=8)** | **WT**  **(n=10)** | | ***Cyp2c70*^-/-^**  **(n=8)** |
| C14:0 | 0.439  (0.410-0.539) | | 0.565  (0.517-0.609) | 0.617  (0.534-0.690) | | 0.971 ***  (0.878-1.001) |
| C16:0 | 8.896  (7.902-12.415) | | 12.645  (10.816-14.440) | 11.924  (10.965-14.991) | | 25.297 ***  (20.808-26.708) |
| C16:1 | 0.140  (0.125-0.172) | | 0.155  (0.145-0.165) | 0.212  (0.184-0.235) | | 0.279 *  (0.244-0.298) |
| C18:0 | 24.956  (19.501-34.726) | | 35.134  (27.226-39.934) | 34.601  (30.030-40.927) | | 81.684 **  (59.825-91.581) |
| C18:1 W7 | 0.667  (0.602-0.851) | | 0.811  (0.664-0.891) | 0.939  (0.828-1.118) | | 1.593 ***  (1.433-1.754) |
| C18:1 W9 | 4.323  (3.599-5.691) | | 5.230  (4.238-5.894) | 6.439  (5.609-7.732) | | 11.686 **  (10.062-12.686) |
| C18:2 W6 | 1.247  (1.059-1.575) | | 1.466  (1.317-1.661) | 1.842  (1.510-2.088) | | 2.989 ***  (2.628-3.114) |
| C18:3 W3 | 0.073  (0.062-0.097) | | 0.091  (0.072-0.105) | 0.106  (0.089-0.122) | | 0.185 ***  (0.163-0.204) |
| C18:3 W6 | 0.168  (0.146-0.193) | | 0.178  (0.165-0.189) | 0.177  (0.148-0.213) | | 0.461 ***  (0.368-0.506) |
| C20:0 | 0.648  (0.521-0.859) | | 0.862  (0.724-0.977) | 0.809  (0.679-0.942) | | 2.107 ***  (1.580-2.255) |
| C20:1 W9 | 0.273  (0.215-0.340) | | 0.318  (0.295-0.353) | 0.373  (0.301-0.430) | | 0.734 ****  (0.596-0.783) |
| C20:4 W6 | 0.307  (0.276-0.339) | | 0.338 *  (0.324-0.383) | 0.421  (0.356-0.452) | | 0.647 **  (0.570-0.697) |
| C22:0 | 0.343  (0.299-0.390) | | 0.467  (0.406-0.516) | 0.388  (0.327-0.426) | | 0.955 ****  (0.845-0.995) |
| C24:0 | 0.209  (0.183-0.232) | | 0.243  (0.216-0.252) | 0.280  (0.222-0.309) | | 0.557 ****  (0.510-0.585) |
| C24:1 W9 | 0.068  (0.065-0.076) | | 0.098 **  (0.092-0.103) | 0.100  (0.075-0.110) | | 0.238 ****  (0.212-0.263) |
| C26:0 | 0.076  (0.075-0.078) | | 0.077  (0.073-0.085) | 0.094  (0.076-0.105) | | 0.162 ***  (0.153-0.177) |
| Total | 42.830  (35.137-58.728) | | 58.639  (47.343-66.704) | 58.963  (51.981-70.055) | | 131.248 ***  (101.621-141.944) |

Data represents median ± interquartile range. P-values represent Mann-Whitney U nonparametric comparisons within genders. *p<0.05, **p<0.01, ***p<0.001, ****p<0.0001. WT, wild-type; WTD, Western-type diet; BW, body weight.


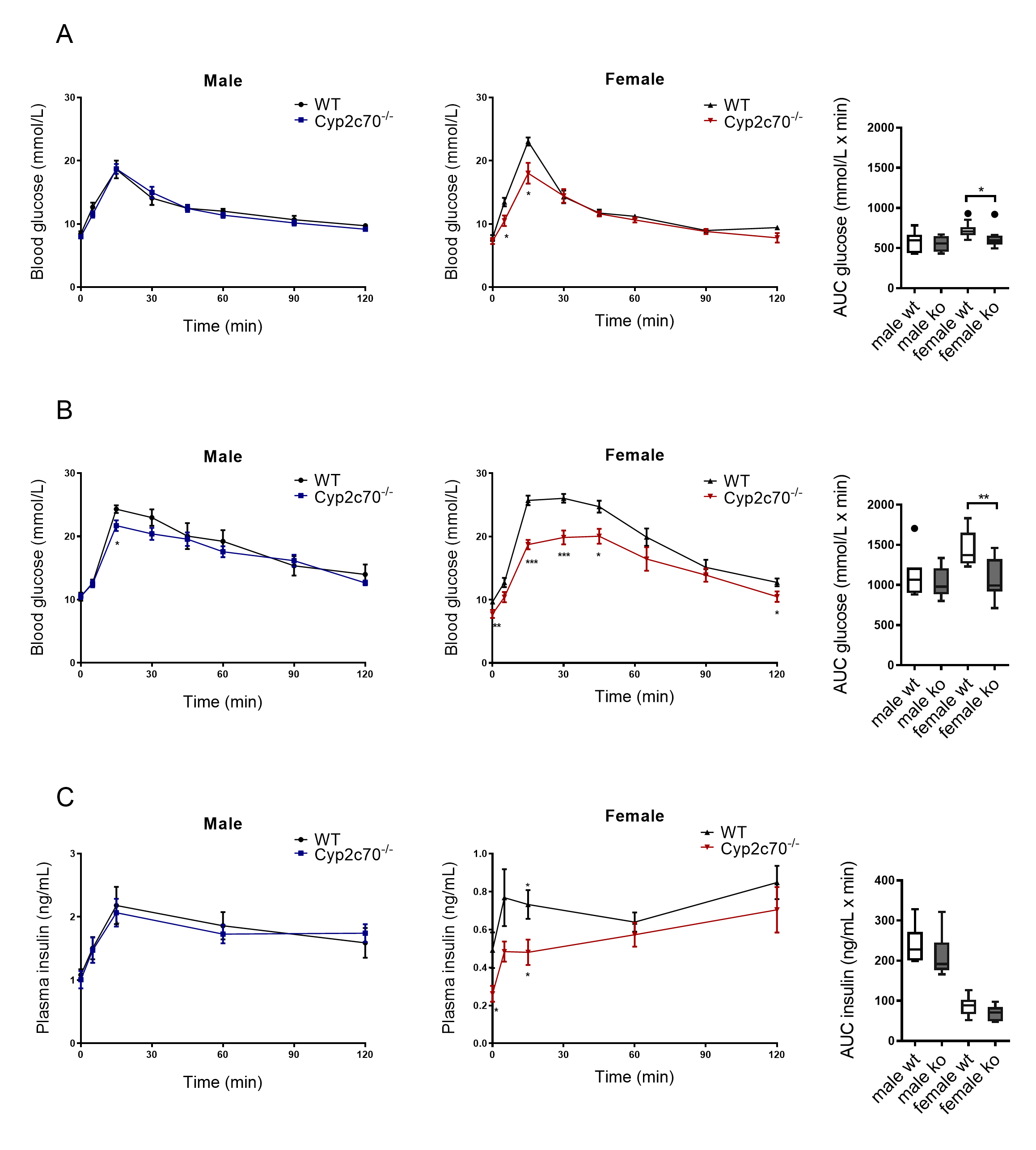


**Supplemental Figure S1. Female, but not male, *Cyp2c70*^-/-^ mice show higher insulin sensitivity than their wild-type littermates after 11 weeks of WTD feeding.**

Blood glucose excursions in male and female *Cyp2c70*^-/-^ mice and their wild-type littermates during oral glucose tolerance tests (OGTTs) performed before (A) and after (B) 11 weeks of WTD feeding. (C) Insulin curves and areas under curve during OGTTs performed after 11 weeks of WTD feeding in male and female *Cyp2c70*^-/-^ mice and their wild-type littermates.

Data are represented as mean ± SD. N=7-10 mice/group. P values represent *p<0.05, **p<0.01, ***p<0.001 by Mann-Whitney U nonparametric comparisons within genders. wt, wild-type; ko, *Cyp2c70*^-/-^ ; WTD, Western-type diet; AUC, area under the curve.

**Supplemental Figure S2. Bile acid composition in plasma in *Cyp2c70*^-/-^ and their wild-type littermates after 12 weeks of WTD.**


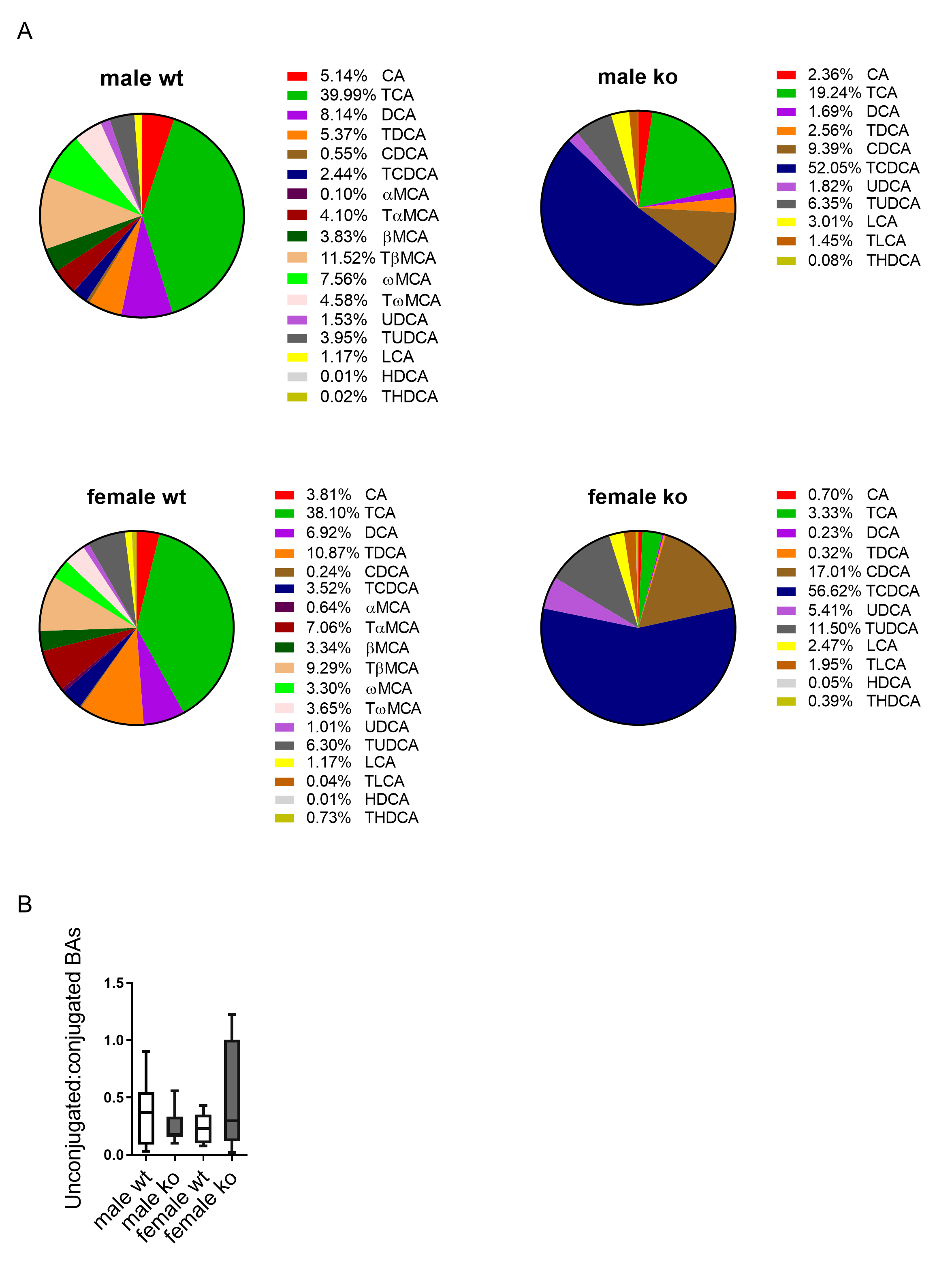


(A) Conjugated and unconjugated BA species in plasma of *Cyp2c70*^-/-^ and wild-type mice after 12 weeks of WTD. Data are represented in pie charts as percentage of total plasma BAs. (B) Ratio unconjugated to conjugated BAs in plasma. Kruskal-Wallis H testing was used for statistical analysis. N=7-10 mice/group. wt, wild-type; ko, *Cyp2c70*^-/-^ ; WTD, Western-type diet; BAs, bile acids.


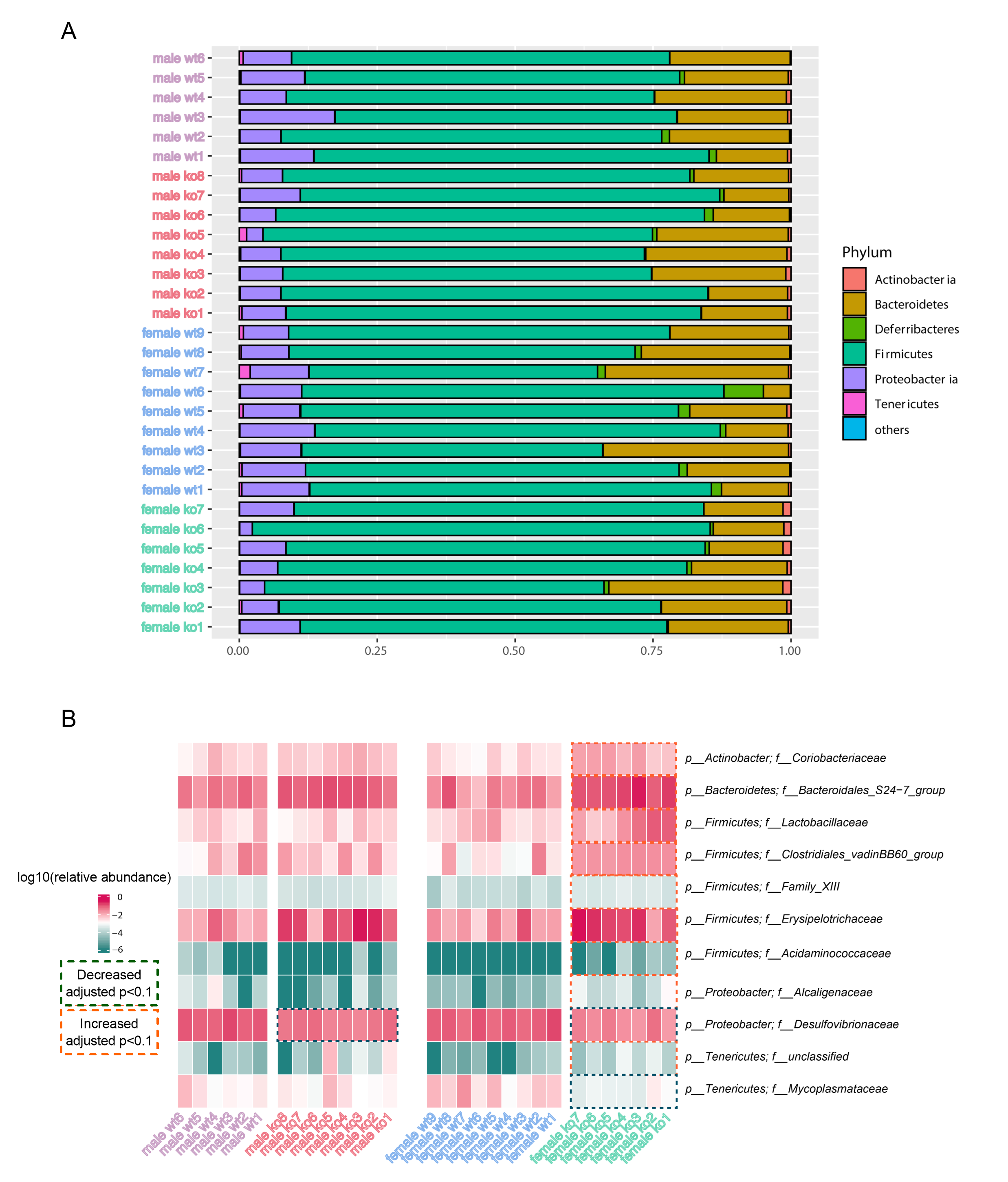


**Supplemental Figure S3. Relative abundance of gut bacteria in *Cyp2c70*^-/-^ and wild-type mice after 12 weeks of WTD.**

(A) Relative abundance of gut bacteria at the phylum level between *Cyp2c70*^-/-^ and wild-type mice. (B) Differential abundance analysis of gut bacteria between *Cyp2c70*^-/-^ and wild-type mice within genders on family level. The rare bacterial families were filtered out if they were presented in less than 5 mice to minimize false discovery. Families are presented in the heat map showing differential abundance between *Cyp2c70*^-/-^ and wild-type mice, detected by Mann-Whitney U nonparametric comparisons within genders. N=6-10 mice/group. The p values were corrected for multiple testing using Benjamini -Hochberg method, with the FDR set at 0.1. wt, wild-type; ko, *Cyp2c70*^-/-^ ; WTD, Western-type diet; p, phylum; f, family; FDR, false discovery rate.


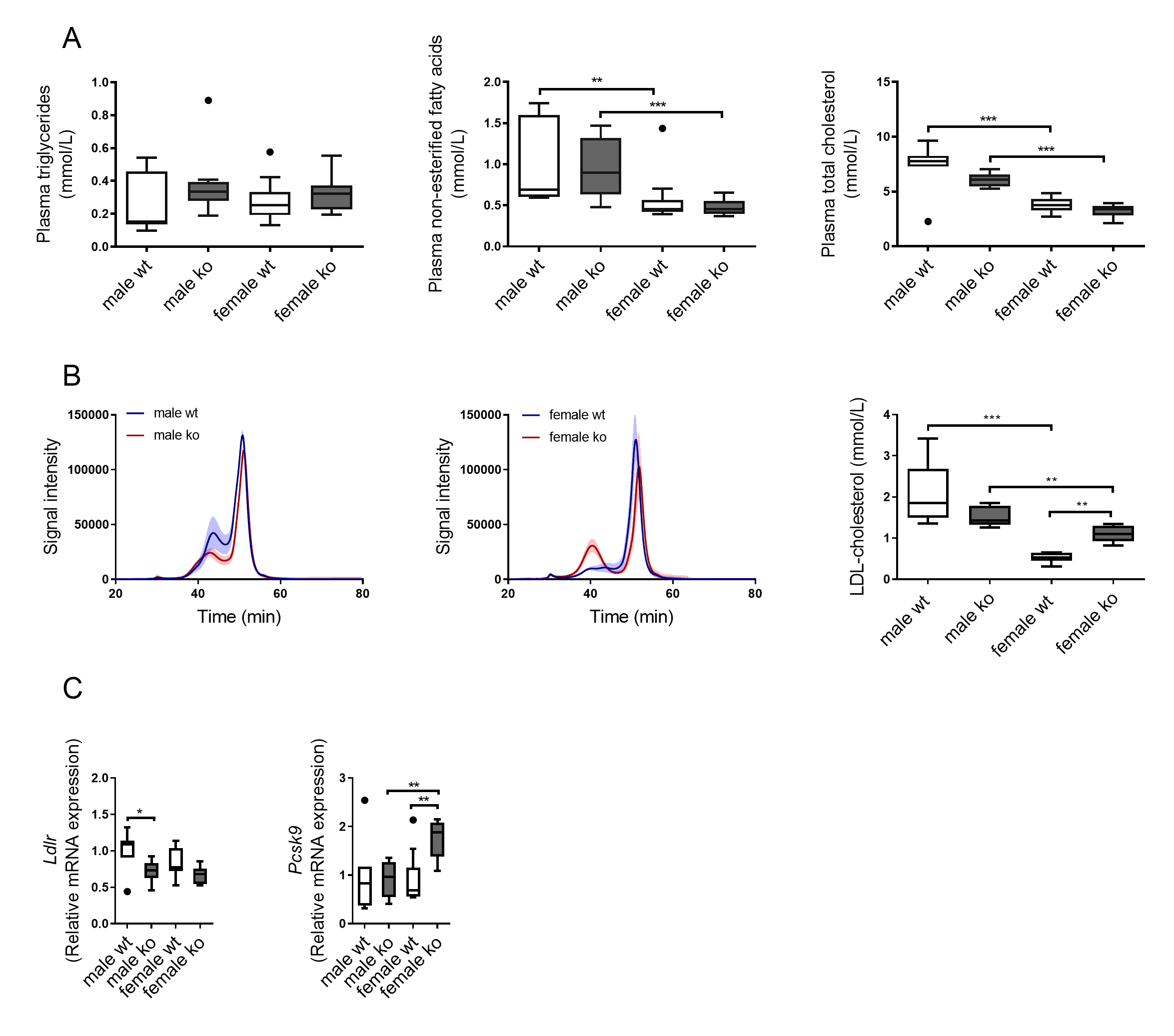


**Supplemental Figure S4. Plasma LDL-cholesterol levels are elevated in female, but not in male, *Cyp2c70*^-/-^ mice compared to wild-type littermates upon feeding WTD for 12 weeks.**

(A) Plasma concentrations of triglycerides, non-esterified fatty acids and total cholesterol in male and female *Cyp2c70*^-/-^ mice and wild-type littermates after 12 weeks of WTD feeding. (B) Plasma lipoprotein profiles obtained by FPLC separation in male and female *Cyp2c70*^-/-^ mice and wild-type littermates after 12 weeks of WTD feeding, represented as mean ± SD for each group, and quantified LDL cholesterol levels per group (right panel). (C) Hepatic mRNA levels of genes involved in LDL clearance by the liver. N=7-10 mice/group. P values represent *p<0.05, **p<0.01, ***p<0.001 by Kruskal-Wallis H testing followed by Conover post-hoc comparisons. wt, wild-type; ko, *Cyp2c70*^-/-^ ; WTD, Western-type diet; *Ldlr*, Low Density Lipoprotein Receptor; *Pcsk9*, Proprotein convertase subtilisin/kexin type 9.


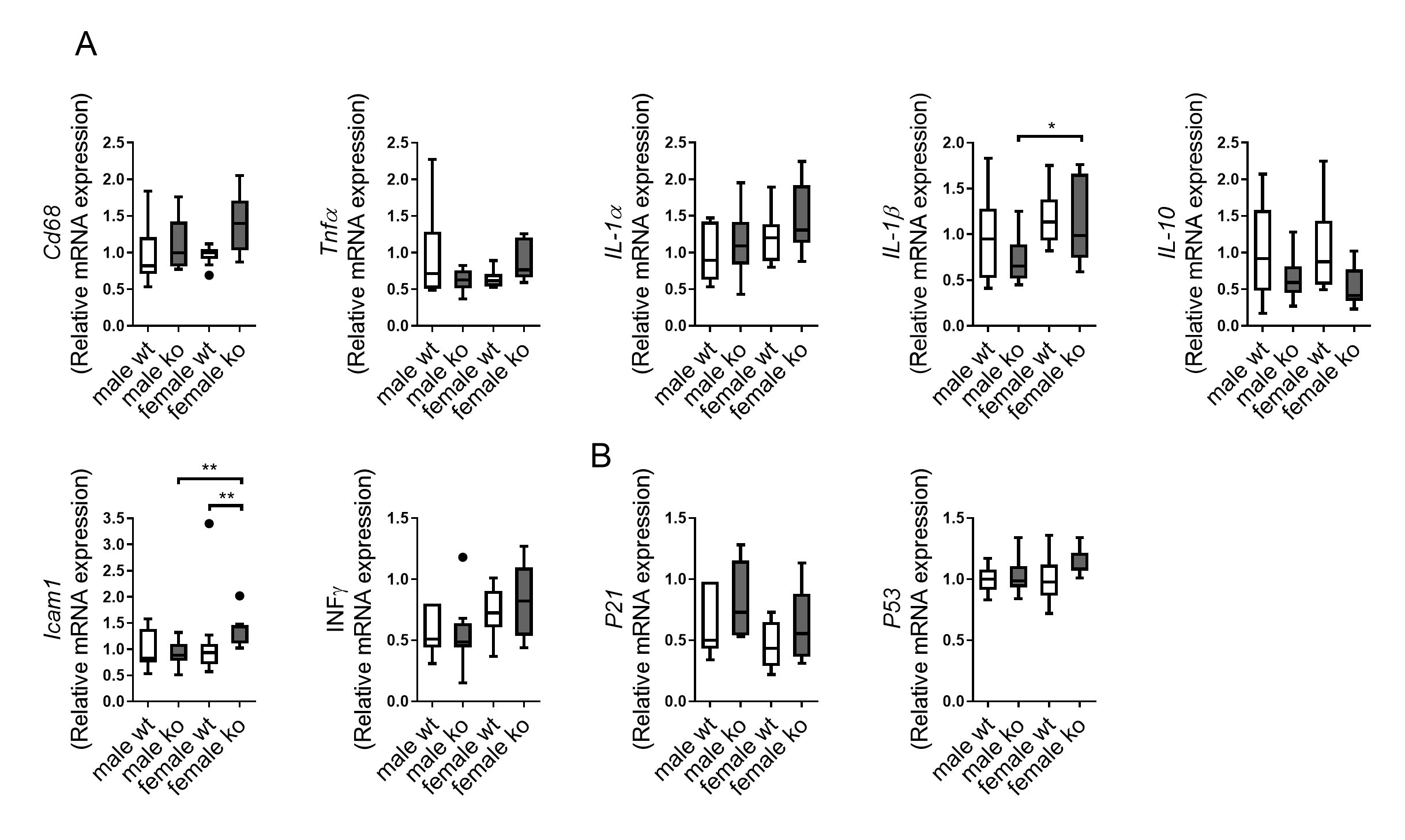


**Supplemental Figure S5. Inflammatory and senescent gene expression in the liver of *Cyp2c70*^-/-^ mice and wild-type littermates.**

Hepatic mRNA levels of genes involved in inflammation (A) and senescence (B) in *Cyp2c70*^-/-^ mice and their wild-type littermates after 12 weeks of WTD. N=7-10 mice/group. Data are presented as Tukey box-and whisker plots. P values represent *p<0.05, **p<0.01, ***p<0.001 by Kruskal-Wallis H testing followed by Conover post-hoc comparisons. wt, wild-type; ko, *Cyp2c70*^-/-^ ; WTD, western-type diet; *Cd68*: cluster of differentiation 68; *Tnfα*, tumor necrosis factor α; *IL-1α*: interleukin 1 alpha; *IL-1β*: interleukin 1 beta; *IL-10*: interleukin 10; *Icam1*, intercellular adhesion molecule 1; INFγ: interferon gamma; *P21*, also known as CDKN1A, CDK-interacting protein 1; *P53*, Tumor protein P53.
